# Modelling tropical forest responses to drought and El Niño with a stomatal optimization model based on xylem hydraulics

**DOI:** 10.1098/rstb.2017.0315

**Published:** 2018-10-08

**Authors:** Cleiton B. Eller, Lucy Rowland, Rafael S. Oliveira, Paulo R. L. Bittencourt, Fernanda V. Barros, Antonio C. L. da Costa, Patrick Meir, Andrew D. Friend, Maurizio Mencuccini, Stephen Sitch, Peter Cox

**Affiliations:** 1 College of Life and Environmental Sciences, University of Exeter, Exeter, UK; 2 College of Engineering, Mathematics and Physical Sciences, University of Exeter, Exeter, UK; 3 Department of Plant Biology, Institute of Biology, UNICAMP, Campinas, Brazil; 4 Instituto de Geosciencias, Universidade Federal do Para, Belem, Brazil; 5 Research School of Biology, Australian National University, Canberra, Australia; 6 School of GeoSciences, University of Edinburgh, Edinburgh, UK; 7 Department of Geography, University of Cambridge, Cambridge, UK; 8 CREAF, Cerdanyola del Valles, Spain; 9 ICREA, Barcelona, Spain

**Keywords:** plant hydraulics, stomatal models, optimality theory, drought, tropical forest

## Abstract

The current generation of dynamic global vegetation models (DGVMs) lacks a mechanistic representation of vegetation responses to soil drought, impairing their ability to accurately predict Earth system responses to future climate scenarios and climatic anomalies, such as El Niño events. We propose a simple numerical approach to model plant responses to drought coupling stomatal optimality theory and plant hydraulics that can be used in dynamic global vegetation models (DGVMs). The model is validated against stand-scale forest transpiration (*E*) observations from a long-term soil drought experiment and used to predict the response of three Amazonian forest sites to climatic anomalies during the twentieth century. We show that our stomatal optimization model produces realistic stomatal responses to environmental conditions and can accurately simulate how tropical forest *E* responds to seasonal, and even long-term soil drought. Our model predicts a stronger cumulative effect of climatic anomalies in Amazon forest sites exposed to soil drought during El Niño years than can be captured by alternative empirical drought representation schemes. The contrasting responses between our model and empirical drought factors highlight the utility of hydraulically-based stomatal optimization models to represent vegetation responses to drought and climatic anomalies in DGVMs.

This article is part of a discussion meeting issue ‘The impact of the 2015/2016 El Niño on the terrestrial tropical carbon cycle: patterns, mechanisms and implications’.

## 1. Introduction

El Niño events contribute to major climatic and ecologic impacts over the Amazon basin [1–4]. Climatically, El Niño events are known to make the climate of most of Amazonia drier and warmer, especially affecting the rainfall patterns in northern Amazonia [4]. This drier climate drives a shift in Amazon forest carbon balance towards a net carbon source to the atmosphere [1,5]. The mechanisms involved in this shift are thought to be related to temperature-induced increases in respiration (particularly soil respiration) and drought-induced decreases in gross primary productivity [3,5,6].

Over the last decade, important advances have been made to improve our understanding of the physiological processes determining plant responses to drought [7,8]. Experimental manipulation and field observations have shown that xylem hydraulic conductance loss is an important mechanism triggering drought-induced plant mortality [9–12]. One of the mechanisms that plants employ to avoid reaching potentially lethal embolism thresholds is the regulation of canopy water potential (*Ψ*_c_) through stomatal control, which creates a coordination between stomatal responses and plant hydraulic conductance losses [13–16]. While process-based models of stomatal functioning based on plant hydraulics have been proposed recently [17–19], most dynamic global vegetation models (DGVMs) rely on empirical drought factors to represent stomatal responses to soil drought [20–23]. These empirical approaches can perform well under many conditions [24–26], but they lack the generality of models that use physiological and ecological theory to predict the responses of vegetation and the global carbon cycle to drier climates [21,27], such as the Amazon climate during El Niño events. In this study we describe and test a new model of stomatal response to drought that is numerically simple enough to implement in a DGVM applicable at large spatial scales, without losing recent theoretical advancements made in the field of plant hydraulics and stomatal optimization theory [17,18].

Our model is based on optimality theory, that is, plant structure and functioning have evolved to maximize efficiencies within the limits of genotypic variation and physico-chemical constraints [28–32]. This principle has been widely used to predict stomatal responses to environmental conditions, starting with Cowan [33] and Cowan & Farquhar [34], where stomata are assumed to maximize carbon assimilation (*A* as carbon mass) while minimizing transpiration (*E* as water mass) over a given time interval (d*t*). This concept can be represented by maximizing the function *A*−*λE* over d*t*. The parameter *λ* represents the marginal carbon cost of water (carbon mass per water mass). This *E*-based optimization approach provides an alternative to empirical models that has been widely used [35–39], such as in Medlyn *et al.* [40] to derive the unified stomatal optimization model (USO). The USO shows the potential of the *E*-based optimization theory to predict stomatal conductance (*g*_c_) responses to environmental drivers [40,41]. However, *E*-based optimization does not account for soil drought effects on *g*_c_, which need to be represented empirically as in Zhou *et al.* [25,26], or with semi-empirical drought factors [36,37].

We represent drought effects on stomatal conductance coupling plant hydraulics with stomatal optimality theory, following the principles outlined in Wolf *et al.* [19] and Sperry *et al.* [18] and using an optimization routine similar to Friend [42]. Sperry *et al.* [18] propose that the costs associated with stomatal opening can be represented as the loss of the plant capacity to transport water, which allows us to replace the need for *λ* with hydraulic traits that determine plant vulnerability to drought-induced embolism. Plant hydraulic traits that determine xylem vulnerability to embolism at the branch-level are currently available for a large number of species of different biomes [43], which makes the hydraulics-based optimization approach particularly attractive for inclusion in ecosystem models.

In this study we validate a stomatal optimization model based on xylem hydraulics (SOX) against scaled-up sap flux observations from an Amazon forest site subject to long-term experimental drought [11,44] and evaluate its predictions against other stomatal models. Subsequently, we investigate how our model predictions differs from empirical drought factors at simulating the response of Amazon forest sites to climatic anomalies during the twentieth century.

## 2. Material and methods

### (a) Model description

**Figure 1.**
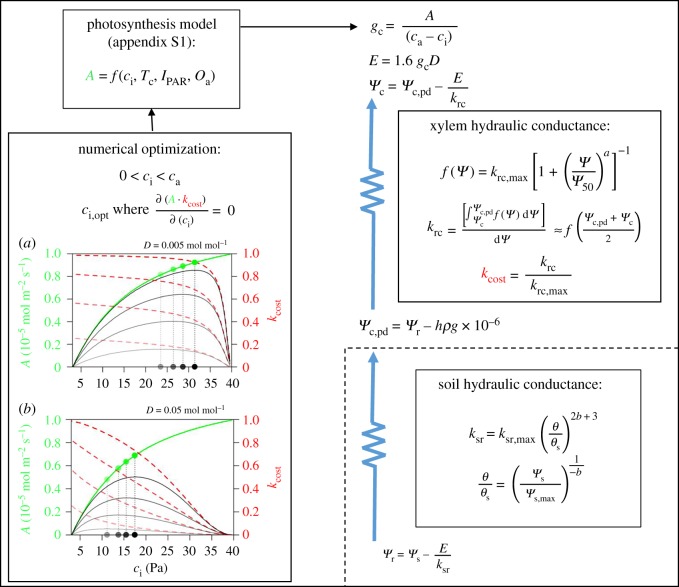
Schematic representation of the stomatal optimization based on the xylem hydraulics (SOX) model. The blue arrows represent the water flow from soil and roots (dashed box) to canopy. The resistor symbols represent dynamic resistances to water flow computed using the equations in the respective boxes. The Collatz *et al.* photosynthesis model (electronic supplementary material, appendix S1) is used to produce the gross carbon assimilation (*A*, green lines in subpanels *a* and *b*) for a set of environmental conditions and initial leaf internal CO_2_ concentration (*c*_i_) value. We use equations (2.1)–(2.3) from the main text to calculate the xylem water potential at the canopy (*Ψ*_c_) asso*c*_i_ated with the given *A* value. The midpoint of the root to canopy *Ψ*_c_ gradient is used to compute the normalized root to canopy hydraulic conductance (*k*_cost_), which represents the cost of stomatal aperture in SOX (red dashed lines in *a* and *b*). The *k*_cost_ and *A* are used to numerically find the optimum *c*_i_ (circles on the *x*-axis of *a* and *b*), at the maximum point of the *A* · *k*_cost_ function (black lines in *a* and *b*). In the subpanels *a* and *b*, we represent the SOX optimization routine at increasing soil drought stress (lighter coloured lines represent lower soil water potential, *Ψ*_s_) at two different levels of atmospheric demand. The optimum *c*_i_ value of the interval evaluated by the SOX routine (0 to atmospheric CO_2_ levels) is used to calculate the optimum *A*, denoted by the circles on the *A*–*c*_i_ curve. The equations in the dashed box represent how changes in soil conductance can be incorporated in SOX by modelling the soil to root hydraulic conductance (*k*_rc_) as a function of *Ψ*_s_ as described in electronic supplementary material, appendix S4. (Online version in colour.)

The SOX model assumes the loss of xylem hydraulic conductance is the main cost associated with stomatal opening. Therefore, we calculate the optimal stomatal conductance for a given set of environmental conditions as the value that maximizes *A* (mol m^−2^ s^−1^) given concurrent hydraulic conductance losses, using a numerical routine similar to the PGEN model [42]. A schematic representation of the model is shown in figure 1. The numerical routine we describe here can be coupled to any photosynthesis model that computes *A* from environmental inputs and the leaf intercellular CO_2_ concentration (*c*_i_, mol mol^−1^). In this study we use the photosynthesis model from Collatz *et al.* [45], following Clark *et al.* [20], described in electronic supplementary material, appendix S1. From an initial value for *A*, we derive the canopy conductance to CO_2_ (*g*_c_, mol m^−2^ leaf s^−1^) and transpiration (*E*, mol m^−2^ leaf s^−1^) as:2.1
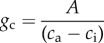
and 2.2
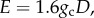
where *c*_a_ is the CO_2_ concentration (mol mol^−1^) in the atmosphere (assumed to be equal to the leaf surface). The leaf-to-air vapour pressure deficit (*D*, mol mol^−1^) is calculated with the assumption that canopy temperature is close to air temperature. These assumptions are justified on the basis that the model implemented in this study is the proof of concept of a scheme designed to be coupled to larger scale models that often employ more detailed calculations of canopy aerodynamical resistance and energy balance (e.g. Best *et al.* [46]). The constant 1.6 is the ratio of water vapour to CO_2_ diffusivities in the air.

The resulting value of *E* is used to calculate the xylem water potential at the canopy (*Ψ*_c_, MPa) using Darcy's Law, assuming steady state conditions (i.e. no contribution of stored water to transpiration):2.3
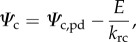
where *k*_rc_ is the root–canopy hydraulic conductance (mol m^−2^ leaf s^−1^ MPa^−1^) and *Ψ*_c,pd_ is *Ψ*_c_ at the pre-dawn which, assuming no night-time transpiration, can be approximated as the root *Ψ* (*Ψ*_r_) adjusted for the canopy height (*h*, m) induced *Ψ* gradient:2.4
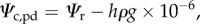
where *ρ* is the water density (997 kg m^−3^), *g* is the Earth's gravitational acceleration (9.8 m s^−2^) and the 10^−6^ converts Pa to MPa. Stored water can contribute significantly to tropical vegetation transpiration [47,48]. However, this contribution is lower during periods of high water stress, when the internal water reserves are depleted, which makes equation (2.3) a reasonable approximation when *Ψ*_c_ is more relevant for our model. The *k*_rc_ in equation (2.3) itself depends on *Ψ*_c_ for its computation as *k*_rc_ declines from its maximum value (*k*_rc,max_) as the xylem pressure (*Ψ*) drops due to cavitation-induced embolism formation [49]. This process can be described with a function such as the inverse polynomial from Manzoni *et al.* [50]:2.5
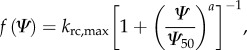
where *Ψ*_50_ is *Ψ* when *k*_rc_ = 0.5*k*_rc,max_ and *a* controls the shape of the function. The empirical relationship between *Ψ*_50_ and *a* from Christoffersen *et al.* [51] described in electronic supplementary material, appendix S2, reduces the plant hydraulic parameters needed in SOX to only *Ψ*_50_, *k*_rc,max_ and *h* used in equation (2.4). SOX is designed as a dynamic model that uses the *k*_rc_ produced at the previous timestep (*k*_rc[_*_t_*_−1]_) to compute the current timestep *Ψ*_c_ and *k*_rc_ via equation (2.3). The purpose of this choice is to facilitate the incorporation of long-term drought effects in *k*_rc_, associated with the incomplete recovery of cavitation [52]. In this study we assume *k*_rc_ recovers instantaneously as *Ψ*_c_ and *Ψ*_c,pd_ increase following a rain event. This might overestimate the fluxes immediately after the dry season, depending on the forest recovery rates from embolism through growth [53,54] or other processes [55,56]. More complex schemes describing partial and gradual *k*_rc_ recovery processes will be explored in future studies.

Sperry & Love [17] and Sperry *et al.* [18] employ the Kirchhoff transform in equation (2.5) to account for the gradual *Ψ* drop along the tree, computing *k*_rc_ as:2.6
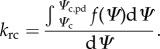


In SOX we represent the gradual *Ψ* drop along the tree using the middle value of the root–canopy gradient (*Ψ*_c,mid_):2.7
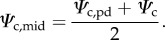


Using *f*(*Ψ*_c,mid_) is numerically simpler than equation (2.6) and provides similar results within a realistic range of *Ψ*_c,pd_ and *Ψ*_c_ (electronic supplementary material, figure S1). The *k*_rc_ produced by *f*(*Ψ*_c,mid_) normalized as a function of *k*_rc,max_, giving *k*_cost_, represents the costs of stomatal aperture in SOX:2.8
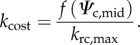
Lower *k*_cost_ implies a higher cost associated with a given level of stomatal aperture. These costs are balanced with *A* using a numerical optimization routine. A detailed discussion of the differences between the cost functions used in SOX and Sperry *et al.* [18] is given in electronic supplementary material, appendix S3.

The SOX optimization routine is implemented in this paper following similar principles to the PGEN model optimization routine [44], which assumes the optimum *g*_c_ can be found where the product between *A* and its unitless drought factor are maximized. In SOX, as *A* and *k*_cost_ are functions of *c*_i_, the optimum *c*_i_, hereafter *c*_i,opt_, for a given set of environmental conditions is found at:2.9
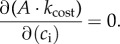


We use an algorithm (see the SOX model code available as electronic supplementary material) to evaluate *c*_i_ over the interval (0, *c*_a_) and find the solution to equation (2.9). The *c*_i,opt_ is used to calculate optimum values of *A*, *g*_c_, *E* and *Ψ*_c_ using the photosynthesis model in electronic supplementary material, appendix S1 and equations (2.1)–(2.3).

Changes in soil hydraulic conductance can also be included in SOX by computing *Ψ*_r_ as a function of soil-to-root conductance as shown in figure 1 and explained in detail in electronic supplementary material, appendix S4. The model evaluations conducted in this study used the simplest version of SOX without the equations from electronic supplementary material, appendix S4 (i.e. assuming *Ψ*_r_ ≈ *Ψ*_s_), unless noted otherwise.

### (b) Model evaluation

The model was written in R (v. 3.4.2; [57]), and the code is available as electronic supplementary material; all the subsequent analyses were also conducted in R. The model responses to environmental drivers were evaluated by holding all meteorological inputs constant at their default values (table 1) and varying a single input at a time. Because equation (2.3) depends on *k*_rc[_*_t−_*_1]_, we run the model at constant environmental conditions for 50 iterations to evaluate SOX instantaneous responses to the environment. This procedure is not necessary when SOX is run as a dynamic model, which is the case when SOX is coupled to a DGVM or in the subsequent model evaluations we conduct in this study.

**Table 1.** Default environmental and plant inputs used in this study.

type	symbol	definition	default value
environmental input	*I*_PAR_	incident photosynthetically active radiation	2 × 10^−3^ mol m^−2^ s^−1^
	*T*_a_	air temperature	20°C
	*D*	vapour pressure deficit	5 × 10^−3^ mol mol^−1^
	*O*_a_	air O_2_ concentration	0.2 mol mol^−1^
	*c*_a_	air CO_2_ concentration	4 × 10^−4^ mol mol^−1^
	*P*_a_	atmospheric pressure	0.1 MPa
	*Ψ*_s_	soil water potential	−0.1 MPa
plant input	ω*	leaf scattering coefficient	0.15
	*V*_cmax25_^a^	maximum Rubisco carboxylation rate at 25°C	5 × 10^−4^ mol m^−2^ s^−1^
	*T*_upp_^a^	high temperature photosynthesis range	40°C
	*T*_low_^a^	low temperature photosynthesis range	10°C
	α*	quantum efficiency	0.1 mol mol^−1^
	*k*_rc,max_	xylem maximum hydraulic conductance	0.01 mol m^−2^ s^−1^ MPa^−1^
	*h*	plant height	20 m
	*Ψ*_50_	xylem water potential when *k*_rc_ = 0.5*k*_rc*,*max_	−2.5 MPa

*Parameters used in the Collatz *et al.* [45] photosynthesis model described in electronic supplementary material, appendix S1.

We evaluated the model capacity to produce realistic predictions of vegetation response to seasonal and experimental soil drought using observations from an evergreen broadleaf tropical forest located in Caxiuanã National Forest in the eastern Brazilian Amazon (electronic supplementary material, figure S3 for site details). We compared the modelled *E* with the stand-scale sap flux data from two 1 ha plots at the site. One of the plots has been subjected to a throughfall exclusion treatment (TFE) since 2001 [11,58], which provides an ideal scenario to test the capacity of SOX to reproduce vegetation response to severe soil drought. Details on sap flux data collection and procedures to scale the data from tree to stand-level can be found in da Costa *et al.* [59]. The meteorological forcing data were collected at the top of a 40 m tower at the site, and the soil moisture data were measured with time-domain reflectometry sensors placed at 0.0–0.3, 0.5, 1 and 2.5 m depth. We used the Clapp & Hornberger [60] equation from electronic supplementary material, appendix S4 to obtain *Ψ*_s_ from the site observations of root mass-weighted soil moisture content (*θ*, m^3^ m^−3^), with the soil hydraulic parameters derived from the soil ancillary data used in the Hadley Centre Global Environmental Model Earth System Model (HadGEM2-ES) [44], which is based on the Harmonized World Soil Database (v. 1.2) [61]. The root biomass profile was modelled using the equations from Best *et al.* [46] assuming soil and root depth were 3 m, which is the default value for broadleaf evergreen tropical trees (BET) used in JULES [20]. We used the site-averaged values of tree hydraulic and physiological data, or the reference JULES values for BET (electronic supplementary material, table S1). Vegetation *k*_rc,max_ was obtained from branch-level xylem specific conductivity (*K*_x_), *h*, the ratio between sapwood area and leaf area (i.e. the Huber value, *h*_v_) and a tapering correction factor calculated following Christoffersen *et al.* [51]; see full description of these calculations in electronic supplementary material, appendix S5. We scale the model predictions from leaf to plot area using the big leaf approach as described in Clark *et al.* [20], with the light extinction coefficient set to the default BET value (0.5) and leaf area index (LAI) fixed at the mean value observed at the site (4.8 m^2^ leaf m^−2^ soil). We consider the use of a fixed LAI in this study is the most parsimonious choice for the purpose of validating our model, considering the small LAI changes observed at the site (standard deviation of 0.5 m^2^ leaf m^−2^ soil).

We compare SOX agreement with observations against a model that uses a drought representation model based on the *β*-function (*β*_fun_) soil drought factor described by Cox *et al.* [24]. A description of this model is given in electronic supplementary material figure S4. We fitted the relationship between *A* and stomatal conductance of water (*g*_w,_ mol m^−2^ s^−1^) predicted by SOX to the unified stomatal optimization model (USO) of Medlyn *et al.* [40], described in electronic supplementary material, appendix S6.

### (c) El Niño simulations during the twentieth century

We compared SOX's sensitivity to drought events with the *β*_fun_ model (electronic supplementary material, figures S4) using meteorological and vegetation hydraulic observations coupled to the modelled soil moisture dynamics of three Amazonian sites (electronic supplementary material, figure S3). We used the CRU-NCEP (v. 4, see supplementary figure S5; N. Viovy, Laboratoire des Sciences du Climat et de l’Environnement, (LSCE), France, 2016, personal communication) 6-hourly meteorological data from 1901 to 2016 to drive our models (see electronic supplementary material, figure S5). These forest sites possess distinct climatic responses to El Niño events (electronic supplementary material, figure S3), represented by the Niño-3 index, which is calculated as the mean sea surface temperature (SST) anomaly from 5°N to 5°S and 150–90°W [62]. Additionally, we used the site-specific monthly soil moisture product from JULES, applied following the TRENDY protocol [27,63], to drive our simulations. The soil hydraulic parameters for each site were obtained from the HadGEM2-ES soil ancillaries [64]. We used the plant inputs given in electronic supplementary material, table S1 to represent the Caxiuanã site. For the Tapajós and Manaus sites, we used the mean plant hydraulic [65] and photosynthetic parameters measured at each site to parameterize the models (electronic supplementary material, table S2), while the other parameters were assumed equal to those of the Caxiuanã site. The vegetation hydraulic trait sampling in each site represents approximately 40, 36 and 15% of the forest basal area for the Caxiuanã, Tapajós and Manaus sites, respectively.

We measured the effects of climatic anomalies on air temperature and atmospheric demand (*T*_a_ and *D*) and soil water availability (*Ψ*_s_) by conducting experiments where we drove the models with the 6-hourly data that correspond to an average year based on the historical climate from the CRU-NCEP dataset (1901 to 2016, electronic supplementary material, figure S5). This procedure eliminates climatic anomalies, such as those associated with El Niño (electronic supplementary material, figure S3). In total we conducted four simulations for each site: Sim1 is the control run using the unaltered CRU-NCEP dataset; Sim2 is the run without anomalies in *T*_a_ and *D*; Sim3 is the run without anomalies in *Ψ*_s_; and Sim4 is without anomalies in any of the previously mentioned variables (*T*_a_, *D* and *Ψ*_s_, see electronic supplementary material, table S3 for summary).

## 3. Results

### (a) Theoretical responses to environment

**Figure 2.**
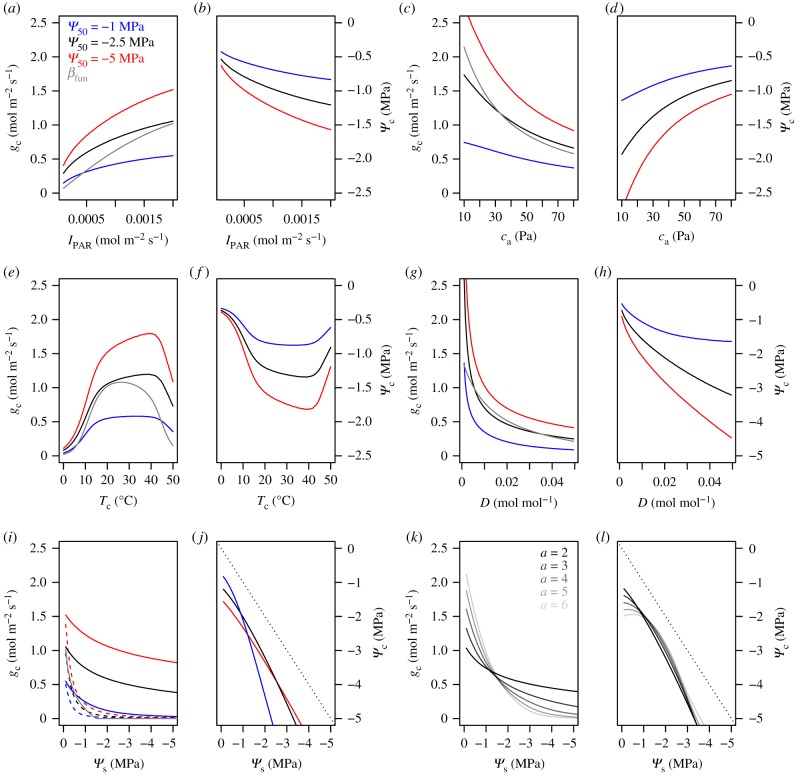
Response curves of stomatal conductance to CO_2_ (*g*_c_, left panels) and canopy water potential (*Ψ*_c_, right panels) to changes in incident photosynthetically active radiation (*I*_PAR_), atmospheric carbon dioxide partial pressure (*c*_a_), canopy temperature (*T*_c_), vapour pressure deficit (*D*) and soil water potential (*Ψ*_s_). All the other environmental inputs and plant inputs were held constant at their default values (table 1), except the parameters of the xylem vulnerability curve represented by different colours. The grey lines are the predictions of the *β*-model described in electronic supplementary material, figure S4. The dashed lines in *i* are the predictions of SOX accounting for changes in soil hydraulics (electronic supplementary material, appendix S4), parameterized with *b* *=* 10, *k*_sr,max_ = 0.1 mol m^−2^ s^−1^ and *Ψ*_s,max_ *=* −0.1 MPa. The dotted line in *j* and *l* is the 1 : 1 line.

SOX predicts that resistance to cavitation produces a stomata behaviour more responsive to changes in incident photosynthetically active radiation (*I*_PAR_), *c*_a_, *T*_c_ and *D*. However, *Ψ*_s_ has a much stronger effect in plants more vulnerable to cavitation (figure 2; electronic supplementary material, figure S6).

The asymptotic stomatal response to *I*_PAR_ (figure 2*a*) is caused by the light-limitation predicted by the photosynthesis model (electronic supplementary material, appendix S1). The SOX predictions represent a hydraulic effect on the plant light response, as plants more resistant to cavitation can sustain light-saturated *g*_c_ 2–3 times higher than the more vulnerable plants. The SOX response to *c*_a_ (figure 2*c*,*d*) is driven by equation (2.2) producing lower *g*_c_ for a given *A* as *c*_a_ increases. The lower intrinsic water use efficiency (i.e. *A*/*g*_c_) at low *c*_a_ is partially compensated in cavitation-resistant plants, which can maintain stomatal aperture with low cavitation costs, reducing the *c*_a_ and *c*_i_ gradient (electronic supplementary material, figure S6). The *T*_c_ response in figure 2*e*,*f* results from the *V*_cmax_–*T*_c_ relationship present in the photosynthesis model (electronic supplementary material, appendix S1), which is more pronounced in plants vulnerable to cavitation. These plants maintain a greater distance to their potential maximum *g*_c_ due to premature, hydraulically-induced stomatal closure.

The SOX response to atmospheric demand (figure 2*g*,*h*) results from the increased *k*_rc_ loss associated with the lower *Ψ*_c_ necessary to maintain carbon assimilation when the atmosphere is drier (i.e. higher *D*, see equations (2.2) and (2.3)). The lower *Ψ*_c_ induces an exponential decline in *g*_c_ as the *c*_i,opt_ shifts closer to 0 (figure 1). This pattern is commonly observed [66–68], and more accentuated in plants with a less negative *Ψ*_50_ owing to their increased cavitation costs as the atmosphere dries (figure 2*g*,*h*). The *β*_fun_ predicts a more gradual stomatal closure in response to *D*, which approximates SOX predictions for *Ψ*_50_ = −2.5 MPa when *D* > 0.02 mol mol^−1^(figure 2*g*).

The SOX response to a drying soil emerges from the same mechanism as its responses to *D*, that is, increased cavitation costs due to the lower *Ψ*_c_ necessary to maintain *A* when *Ψ*_s_ is low. SOX predicts a more gradual decline in *g*_c_ in response to drying soil when compared with the *β*_fun_ model (figure 2*i*,*j*). The *β* model predicts *g*_c_ = 0 when *Ψ*_s_ = −1.5 MPa and *θ* = *θ*_w_ (electronic supplementary material, figure S4; figure 2*i*,*j*), whereas SOX predicts that even plants very vulnerable to cavitation (*Ψ*_50_ = −1 MPa) still have 30% of their maximum *g*_c_ at the same *Ψ*_s._ Accounting for changes in *k*_sr_ with equations in the electronic supplementary material, appendix S3 makes *g*_c_ more responsive to *Ψ*_s_, affecting particularly plants more resistant to cavitation. At *Ψ*_s_ = −5 MPa, even plants with *Ψ*_50_ = −5 MPa will have dropped to 1% of their maximum *g*_c_, whereas in the model that assumes *Ψ*_r_ ≈ *Ψ*_s_ the plant would still have 58% of its maximum *g*_c_. Using a steeper vulnerability curve (higher *a* from equation (2.5)) also greatly increases the plant sensitivity to soil drought (figure 2*k*,*l*). The *a* also affects the *Ψ*_c_ response to *Ψ*_s_, which is linear when *a* is low, but higher *a* produces a more stable *Ψ*_c_ at high soil moisture (figure 2*j*,*l*).

### (b) Model evaluation

**Figure 3.**
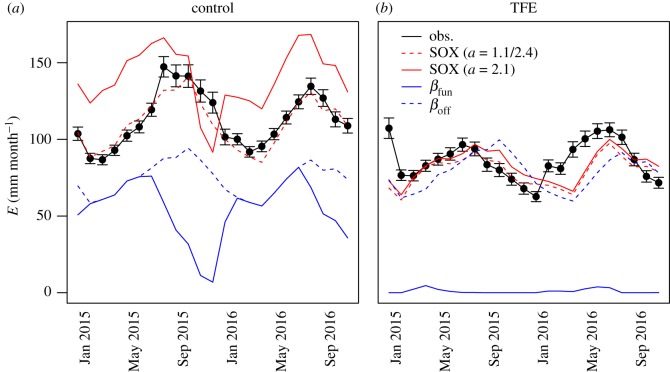
Evaluation of simulated monthly forest transpiration (*E*) against measured forest *E* (black) at the control (*a*) and throughfall exclusion (TFE, *b*) plots in the Caxiuanã National Forest. The dashed red lines are SOX predictions with the shape parameter of the vulnerability curve optimized for each plot, whereas the continuous lines have a single *a* for both plots, calculated as a function of the site *Ψ*_50_ (electronic supplementary material, table S1), following Christoffersen *et al.* [51] (electronic supplementary material, appendix S2). The *β*_off_ model (dashed blue lines) is identical to the *β*_fun_ model (solid blue line) described in electronic supplementary material, figure S4, but the soil drought factor *β* is set to 1. The error bars show 2× standard error.

The SOX predictions agree with the *E* observed at both plots in Caxiuanã more consistently than the alternative *β*_fun_ models (figure 3; electronic supplementary material, figure S7). We were able to approximate the sap flux at both the control and the TFE plot, even though we made the simplifying assumption that the vegetation of both plots was identical (electronic supplementary material, table S1) and used the relationship from Christoffersen *et al*. [51] to estimate the shape parameter of the vulnerability curve (*a*), which predicts *a* = 2.1. Optimizing *a* to the observations of each plot produces a very high agreement on the control plot (*a* = 2.4; *R* = 0.94) and a strong agreement on the TFE plot (*a* = 1.1; *R* = 0.44). Accounting for changes in *k*_sr_ (electronic supplementary material, appendix S4) allows us to improve even further the agreement between SOX predictions and observations in the TFE plot (*R* = 0.5). SOX can also reproduce well the observed seasonal fluctuations in *Ψ*_c_ (electronic supplementary material, figure S8). The *β*_fun_ model greatly overestimates the soil moisture effects, leading to excessive stomatal regulation in the control treatment (*R* = −0.3) and almost complete stomatal closure in the TFE (figure 3*b*; electronic supplementary material, figure S8). A model that ignores soil moisture effects (*β*_off_) can fit the control plot data (*R* = 0.94) but cannot capture the seasonality in the TFE plot (*R* = 0.11).

**Figure 4.**
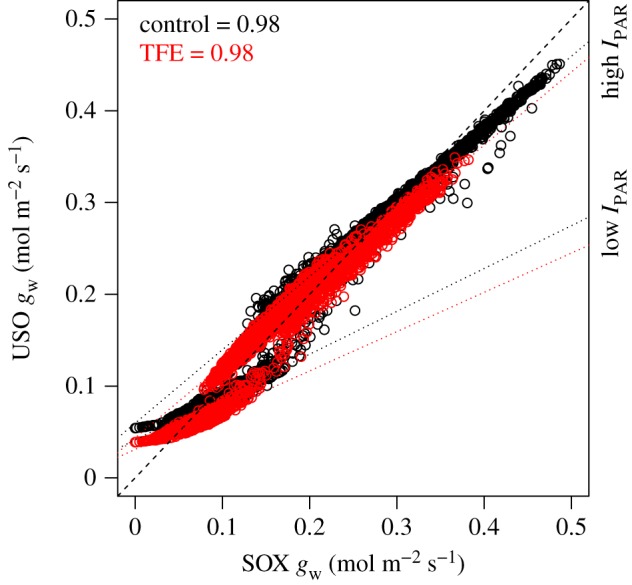
Comparison between the unified stomatal optimization model (USO) and SOX. Red circles are the model predictions from the throughfall exclusion treatment (TFE) in the Amazon forest (Caxiuanã National Forest), black circles are the control treatment. The dotted lines are derived from linear regressions fitted to the data at high (greater than 10^−4^ mol m^−2^ s^−1^) and low (less than 10^−4^ mol m^−2^ s^−1^) incident photosynthetically active radiation (*I*_PAR_) levels. The dashed line is the 1 : 1 relationship.

The relationship between *g*_w_ and *A* predicted by SOX agrees with the Medlyn *et al*. [40] USO model under high *I*_PAR_ (figure 4), and produces estimates of *g*_1_ and its response to *Ψ*_c,pd_ (electronic supplementary material, table S4; figure 4) within the range observed for tropical trees in other studies [26,69]. Deviations from the 1 : 1 line occur at low *g*_w_ and are associated with low *I*_PAR_ periods. These deviations are present even if we set the minimum conductance parameter from USO, *g*_0_, to 0. Therefore, the SOX *A–g*_w_ relationship implies a dependency of the water marginal carbon costs (related with the USO parameter *g*_1_, see electronic supplementary material, appendix S6) on the light regime that is not present in USO. The *g*_1_ predicted by USO is lower at the TFE plot than in the control plot (electronic supplementary material, table S4), indicating that SOX predicts a higher water carbon cost at the TFE. This pattern cannot be observed with the USO parameters estimated from the *β* model's output (electronic supplementary material, table S4).

### (c) El Niño predictions during the twentieth century

**Figure 5.**
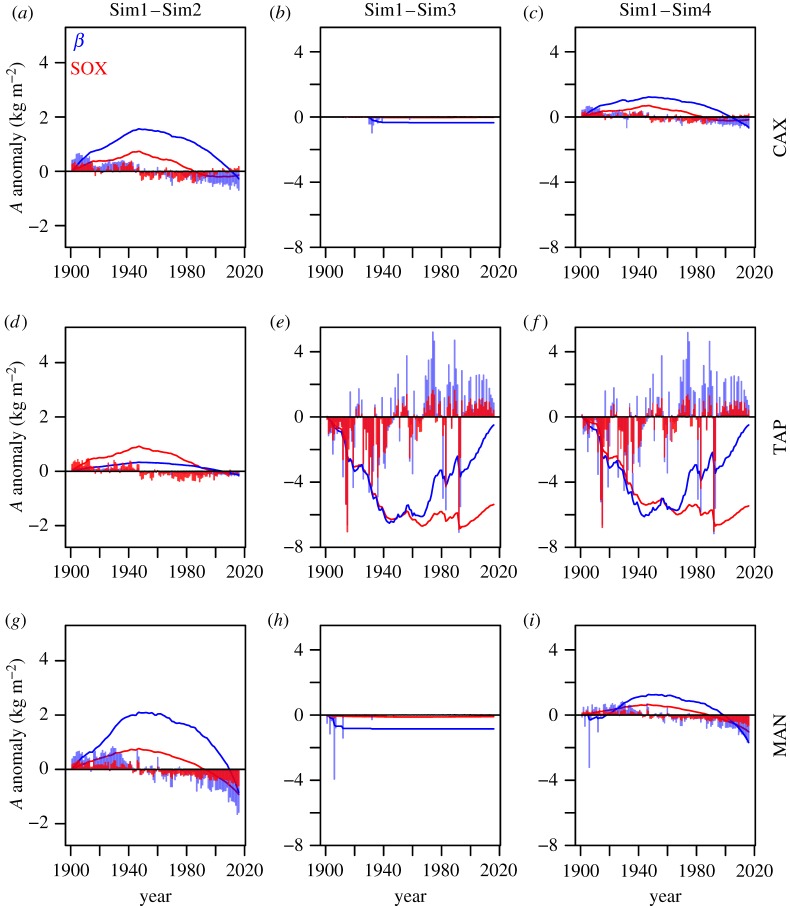
Canopy gross carbon assimilation (*A*) differences between simulations driven with the meteorological data from the unaltered CRU-NCEP dataset (Sim1) and the simulations without climatic anomalies in atmospheric temperature and vapour pressure deficit (Sim2), and soil water potential (Sim3), and without anomalies in any of the previously mentioned variables (Sim4). The bars are the annual *A* anomalies multiplied by 10 to facilitate visualization, and the lines are the accumulated *A* anomalies. The SOX predictions are in red and the *β*-function model in blue. A positive value indicates that climatic anomalies increase *A*, whereas a negative value indicates a negative effect of climatic anomalies on *A*.

Tapajós was the only site where JULES predicted significant soil drought, which could be particularly intense in El Niño years (electronic supplementary material, figures S3, S5 and S9). At this site, the *β*_fun_ model is oversensitive to soil drought, strongly limiting *A* (electronic supplementary material, figure S10) in a similar way to what is observed in figure 3. The *β*_fun_ model is also more sensitive to soil drought anomalies, as shown by the greater interannual variability between Sim1 and Sim3 in Tapajós (figure 5). Both models produce a similar magnitude of negative responses to soil drought anomalies of *ca* −0.7 kg C m^−2^, but *β*_fun_ predicts that *A* can rise by up to 0.52 kg C m^−2^ in years when the soil is more humid than usual, while SOX predicts a maximum increase of 0.16 kg C m^−2^ yr^−1^ (figure 5*e*). This divergence amplifies over the years, leading to the cumulative effect of soil drought anomalies in Tapajós predicted by the *β*_fun_ model being −0.49 kg C m^−2^ after 115 years, while SOX predicts a strong negative cumulative effect of −5.37 kg C m^−2^.

The effect of atmospheric anomalies is comparatively small in Tapajos (figure 5*d*,*e*), but is the dominant effect in Caxiuanã and Manaus (figure 5*a–c*,*g–i*). Atmospheric anomalies tended to increase *A* until *ca* 1950, with *β*_fun_ predicting a maximum effect of accumulated anomalies of 2.1 kg C m^−2^ in 1947 at Manaus, whereas SOX predicts only 0.76 kg C m^−2^ at the same year (figure 5*g*). The increase of frequency and magnitude of positive climatic anomalies in the second half of the twentieth century (electronic supplementary material, figure S9) had a detrimental effect on forest *A*, particularly strong in Manaus. The *β*_fun_ model predicts that at the end of the 115 years climatic anomalies would reduce forest *A* by 0.85 kg C m^−2^, while SOX predicts a reduction of 0.92 kg C m^−2^. The responses of Caxiuanã to climatic anomalies are similar to Manaus but less pronounced, with an overall cumulative effect of climatic anomalies of −0.62 kg C m^−2^ according to *β*_fun_ and −0.15 kg C m^−2^ by SOX (figure 5*c*).

## 4. Discussion

Our results show that a xylem hydraulics-based stomatal optimization scheme can produce realistic stomatal responses to environmental variables (figure 2), being able to predict the observed responses of a tropical forest to seasonal, and even severe experimentally-induced soil drought (figure 3). This finding complements recent studies that have established the theoretical basis for a hydraulically-based model of plant stomatal responses to drought [17,18], and supports the recent findings of Anderegg *et al*. [70], showing the potential of xylem hydraulics-based optimization approaches to simulate the responses of tropical forests to drought. The SOX predictions agree with other models based on the optimality theory, such as the USO, under most circumstances (figure 4). However, SOX predictions are considerably different from the drought factor approach, represented here by the *β*_fun_ model (figures 4 and 5; electronic supplementary material, figure S4). The drastic differences that emerge from long-term simulations between SOX and the *β*_fun_ model (figure 5) highlight the importance of using a more mechanistic plant hydraulic representation to simulate the effects of climatic anomalies, such as El Niño, on forest carbon and water fluxes.

The drastic divergence between the *β*_fun_ predictions and observations found in our study (figure 3) could be partly explained by the choice of using soil moisture data to drive our simulations. The *β*_fun_ model and other empirical drought factors used in DGVMs are often coupled to a soil hydrology scheme [20,46,71,72]. The influence of plant transpiration on soil moisture dynamics could attenuate the extreme soil drought responses we observed (figure 5). However, other studies show that even when soil hydrology is accounted for, *β*_fun_ might still overestimate soil drought responses [73]. The approach we adopted can be considered a conservative test of the model capability to predict forest transpiration, as no assumptions were made modelling the soil water dynamics.

### (a) Generality and limitations of SOX

Our model is designed to be coupled to large-scale ecosystem models such as DGVMs, and therefore its performance depends on the coupled routines representing vegetation processes (e.g. photosynthesis, canopy energy balance, and phenology), soil hydrology and atmospheric processes. For this study we assumed constant leaf area over time when scaling from leaf-level to plot-level in figure 3, as the LAI variation at this site is relatively small. However, phenology schemes [20,74] should be easily integrated with SOX; in addition, our model opens the possibility for plant hydraulics-driven phenology schemes. Linking vegetation phenology to drought responses is a much-needed functionality in many ecosystem models [21,75], and could further improve how SOX represents vegetation responses to extreme drought (figure 3*b*). The hydraulic processes represented by SOX also open up the possibility for a more explicit representation of drought-induced mortality in DGVMs. The thresholds of hydraulic conductance loss associated with increased risks of plant mortality, thought to be close to 0.5*k*_rc,max_ for gymnosperms [12] and 0.12*k*_rc*,*max_ for angiosperms [9,10], can be linked from the SOX output into a DGVM module that controls vegetation demographic processes, such as the TRIFFID module currently used in JULES [74].

The good performance of the simplified SOX implementation we show in this study, which is comparable even to that of more detailed models previously used on the site [51,76], illustrates the parsimony of the xylem hydraulics-based optimization approach. Our model evaluation at the Caxiuanã TFE plot shows that accounting for soil hydraulic conductance loss is an important step for reproducing long-term drought effects (electronic supplementary material, figure S7). These results complement previous work made at the site [76], showing that even after over a decade of experimental drought, soil hydraulic conductance loss remains an important driver for forest response to drought. Even accounting for changes in soil conductance, the performance of SOX in figure 3*b* shows that there is room for improvement in how we model long-term drought in SOX. Together with phenological responses to soil drought mentioned above, legacy effects of cavitation [52] could be an important mechanism driving the TFE plot responses. The SOX treatment of *k*_rc_ in equation (2.3) makes it simple to incorporate the processes determining the recovery of *k*_rc_ by the plant.

The accuracy of our model predictions requires further testing against observations from other ecosystems and plant functional types (PFTs). The agreement of our model predictions with data depends on the two main theoretical assumptions of optimality theory being satisfied: (1) that it is physiologically possible for plant stomata to operate close to the SOX definition of optimum, and (2) the optimization criterion used in SOX can be strongly linked to plant fitness [29–32]. Plant stomata have been often observed to function close to a theoretical optimum [34,38–41,77], but deviations from this behaviour have also been observed [78,79]. These departures can be interpreted as consequences of physical and biochemical limitations on stomatal reaction times [80]. These effects should be more conspicuous at short time-scales and in PFTs with slower stomatal responses, such as gymnosperms [79]. Other mechanisms that have been proposed to cause stomatal departure from a theoretical optimum include non-stomatal limitations to *A*, such as a reduction of Rubisco activity [26,35] and mesophyll conductance [81].

The second SOX assumption concerns our optimization criterion as the maximization of the cost-regulated carbon assimilation product (*A · k*_cost_). The optimality theory replaces the need for detailed physiological parameterization, with evolutionary assumptions that depend on the impact of specific processes and structures on the fitness of organisms [29–32]. The link between *A* and plant fitness is clear, as the reproductive success of a plant depends on its energetic investment in reproductive tissues over its lifespan [82], and in tissues necessary for survival and acquisition of resources other than carbon. The cost term in SOX, represented by xylem hydraulics dysfunction, implies that the complete loss of hydraulic conductance (i.e. *k*_cost_ = 0) would be associated with plant mortality, which represents the ultimate fitness cost [18,19,82]. There is substantial evidence that high levels of xylem cavitation-induced embolism are in fact associated with plant mortality [9–11], which corroborates this assumption. Even non-lethal loss of hydraulic conductivity should be detrimental to plant fitness, as recovery of hydraulic conductivity through construction of new vessels [53,54,83], or through active refilling of embolized vessels [84–87], requires carbon investment, which would necessarily detract from plant tissue growth and reproductive investments. Differences in plant capabilities of recovering hydraulic conductivity, be it through refilling or through the construction of new vessels, imply that a given level of hydraulic damage predicted by the xylem vulnerability function might not fully represent the costs of stomatal opening, as the long-term carbon balance impact of embolism are not explicitly represented. Even though the normalized xylem vulnerability-based cost function we use here represents a satisfactory first approximation, an appropriated weighting of the carbon costs associated with the recovery of hydraulic conductivity [54,56] might be a necessary theoretical development to improve the generality and accuracy of xylem hydraulics-based optimization models.

### (b) Agreement with alternative drought-representation schemes

The relationship between *g*_w_ and *A* predicted by SOX agrees well with that of the USO model from Medlyn *et al.* [40], which reflects the agreement between the different optimization principles underlying each model. The USO assumes stomata maximize the mass of carbon gain per mass of water lost (i.e. *A*−*Eλ*), while SOX maximizes the fraction of xylem lost per mass of carbon assimilated (*A · k*_cost_). The association between these principles can be interpreted as a result of the dependency between *E* and *k* loss (equations (2.3) and (2.5)). As high *E* has no direct detrimental effect on plant fitness, its association with plant hydraulics provides the necessary theoretical link between *E* and plant fitness to satisfy the fundamental assumption of optimality theory [28–32].

Xylem hydraulics-based optimization models have the advantage of combining stomatal responses to *D* and *Ψ*_s_ using a few hydraulic parameters (table 1) that are currently widely available [43]. The difference between our integrated drought representation and approaches usually employed in DGVMs that rely on combining two empirical/semi-empirical functions [20,24,72] is highlighted in the long-term simulations and their responses to climatic anomalies (figure 5). The carbon assimilation in Caxiuanã and, especially, in Manaus was dominated by atmospheric anomalies, as there was little soil drought in the driving data used for this experiment. The soil moisture data used to drive these simulations were the product of large-scale JULES simulations and meteorological datasets (0.5° × 0.5° resolution), which explains their contrast with the environmental data collected at the site that was used to drive the model in the evaluation against sap flux data from Caxiuanã (figure 3). Atmospheric demand is an important driver of vegetation carbon and water fluxes [88], and a more likely mechanism driving Amazon forest responses to climatic anomalies than soil water stress, as the latter often requires multiple years of sustained rainfall reduction to produce a significant response in tropical forests [11,44,59].

Tapajós was the only site with a significant interannual *Ψ*_s_ variability (electronic supplementary material, figure S3), and it was the site where the divergences between the *β*_fun_ model and SOX were largest (figure 5; electronic supplementary material, figures S9 and S10). The *β*_fun_ excessive soil moisture response and highly variable response to climatic anomalies reflect the steep gradient between the critical and wilting points of the *β*_fun_ equation (electronic supplementary material, figure S4), producing a stronger decline in *g*_c_ in response to soil drought than SOX, especially for plants more resistant to cavitation and with lower *a* value (figure 2*i*,*k*). Other studies have also shown that the excessive stomatal regulation produced by the *β*_fun_ produces divergences between model predictions and seasonal GPP patterns in Tapajós [73]. The large discrepancy between the two models, especially over the last 50 years, indicates that tropical forest sites exposed to soil water limitations during El Niño years might have stronger responses to climatic anomalies than can be captured by models based on empirical drought factor schemes.

## 5. Conclusion

Our stomatal optimization model, SOX, provides a simple but theoretically robust approach to simulate tropical forest responses to drought, capable of reproducing the effects of even severe experimental droughts. A process-based representation of atmospheric and soil drought responses is essential for the unbiased simulation of tropical forest responses to El Niño-style climatic anomalies. Improving the representation of plant hydrodynamics is a priority for the current generation of ecosystem models [21–23,27]. The flexibility, relative simplicity and small number of parameters required by SOX make it an attractive candidate to be used in large-scale modelling of tropical forest responses to climate change and extreme climatic anomalies. More studies are necessary to assess the generality of our approach in distinct PFTs and environments, and there is a potential need to incorporate additional mechanisms, such as processes involved in the recovery of hydraulic conductance, hydraulically-driven phenological changes, and mortality.

## Supplementary Material

Appendix

## Supplementary Material

SOX code

## Supplementary Material

BETA code

## Data accessibility

The JULES soil moisture output used in this study as well as the meteorological driving data were obtained from the TRENDY project. The full TRENDY dataset (http://dgvm.ceh.ac.uk/index.html) is available, subject to the individual modelling group approval, via a request to S.S. (s.a.sitch@exeter.ac.uk). The sap flux data used for model validation is published in da Costa *et al.* [59]. The R code for the models used in this paper and the plant input data for each site used in this study are available as electronic supplementary material.
