# Supplementary material for: Modelling tropical forest responses to drought and El Niño with a stomatal optimization model based on xylem hydraulics
Source: Philos Trans R Soc Lond B Biol Sci. 2018 Oct 8;373(1760):20170315. doi: 10.1098/rstb.2017.0315 (PMC6178424; doi:10.1098/rstb.2017.0315)
Supplement: Appendix [file rstb20170315supp1.docx]

Modelling tropical forest responses to drought and El Niño with a stomatal optimization model based on xylem hydraulics

APPENDIX CONTENT

*Supplementary Notes:*

S1. Photosynthesis model description*…………………………………………………………………….*2

S2. Calculation of the shape parameter of the xylem vulnerability curve 3

S3. SOX cost function details 3

S4. Changes in soil hydraulic conductance 3

S5. Calculation of plant maximum hydraulic conductance 4

S6. Description of the Unified Stomatal Optimisation model 5

*Supplementary Tables:*

S1. Plant inputs used for the Caxiuanã National forest site*…………………………………………….*6

S2. Plant inputs used for the Manaus and Tapajós forest sites 6

S3. Description of the simulations conducted at the three amazon sites 6

S4. Parameters from the Unified Stomatal Optimisation model. 7

*Supplementary Figures:*

S1. Comparison of equations 5 and 6 to compute the normalised root to canopy hydraulic conductance....…………………………………………….*…………………………………..……….8*

S2. Effect of different critical values of hydraulic conductance for the computation of the normalised root to canopy hydraulic conductance 9

S3. The three Amazon forest sites used in our study. 10

S4. Description of the β-function model used in the study………………………………………11

S5. Environmental data used to drive the model for the long-term simulations for the three Amazonian sites used in this study.………………………………………………………...……12

S6. Theoretical relationships between intracellular to atmospheric CO_2_ and environmental variables 13

S7. Taylor diagram using the monthly evaporation predictions for the Caxiuanã site. 14

S8. Minimum daily *β* values predicted by the β-function model and the minimum daily normalised root to canopy hydraulic conductance from SOX on the Caxiuanã forest simulation 15

S9. Anomalies in air temperature, vapour pressure deficit and soil water potential at each study site 16

S10. Annual canopy gross carbon assimilation predicted by SOX and by the β-function model for the three sites used in this study 17

**Supplementary Notes**

**Notes S1.** *Photosynthesis model description*

We model vegetation carbon assimilation (*A*; mol m^-2^ s^-1^) using the C3 photosynthesis model of Collatz *et al.* [1] as described by Clark *et al.* [2]. This model calculates *A* as the smoothed minimum of three potentially-limiting photosynthetic rates: rubisco-limited rate (*W*_c_), light-limited rate (*W*_l_) and transport-limited rate (*W*_e_).

The Rubisco limited photosynthesis rate (*W*_c_) is given by:

$W_{c}=V_{cmax}\frac{c_{i}-\Gamma}{c_{i}+K_{c}(1+{O_{a}}/{K_{o}})}$

Where *V*_cmax_ is the Rubisco maximum carboxylation rate (mol m^-2^ s^-1^), *c_i_* is the leaf internal CO2 partial pressure (Pa), *O*_a_ is the oxygen partial pressure in the atmosphere (Pa), Γ is the photorespiration compensation point (Pa), and *K*_c_ and *K*_o_ are Michaelis-Menten constants for CO_2_ and O_2_, respectively. *V_cmax_* is calculated as:

$V_{cmax}=\frac{V_{cmax}25-2^{0.1(T_{c}-25)}}{\left[ 1+e^{0.3(T_{c}-T_{upp})} \right]\left[ 1+e^{0.3(T_{low}-T_{c})} \right]}$

Where *T*_c_ is the canopy temperature (C), assumed to be equal to *T*_a_ in our study. The parameters *T*_upp_ and *T*_low_ define the optimal temperature range for Rubisco activity, and V_cmax25_ is V_cmax_ at 25 °C.

The photorespiration compensation point, Γ is calculated as:

$$\Gamma=\frac{O_{a}}{2\tau}$$

where τ is:

$$\tau=2600[{0.57}^{0.1\left( T_{c}-25 \right)}]$$

*K*_c_ and *K*_o_ are given by:

$$K_{c}=30[{2.1}^{0.1\left( T_{c}-25 \right)}]$$

and:

$$K_{o}=3\times{10}^{4}[{1.2}^{0.1\left( T_{c}-25 \right)}]$$

The light limited photosynthesis rate (*W*_l_) is given by:

$$W_{l}= \alpha(1-\omega)I_{par}\left( \frac{c_{i}-\Gamma}{c_{i}+2\Gamma} \right)$$

where α is the quantum efficiency of photosynthesis (mol mol^-1^), ω is the leaf scattering coefficient (unitless) and *I*_par_ the photosynthetically active radiation incident on the leaf (mol m^-2^ s^-1^).

The transport limited photosynthesis rate (*W*_e_) is given by:

$$W_{e}=0.5V_{cmax}$$

The smoothed minimum of the three rates (i.e. *A*) is calculated by solving for the smaller root of each of the following quadratic equations:

$$\beta_{1}W_{p}^{2}-W_{p}\left( W_{c}+W_{l} \right)+W_{c}W_{l}=0$$

$$\beta_{2}A^{2}-A\left( W_{p}+W_{e} \right)+W_{p}W_{e}=0$$

where *W*_p_ is the smoothed minimum of *W*_c_ and *W*_l_*,* β_1_ and β_2_ are the co-limitation coefficients, set to 0.83 and 0.93, respectively [2].

**Notes S2.** *Calculation of the shape parameter of the vulnerability curve*

We calculate the shape parameter of the vulnerability curve (*a*) in function of the xylem Ψ_50_ using the empirical relationship from Christoffersen *et al.* [7]. The slope of the linear portion of the xylem vulnerability function (*s_x_*, MPa^-1^) is related to Ψ_50_ as:

$$s_{x}=65.15{(-\Psi_{50})}^{-1.25}$$

and *a* from the equation 5 in the main text can be calculated as:

$$a=\frac{-4s_{x}}{100\Psi_{50}}$$

**Notes S3.** *SOX cost function details*

The cost function used in SOX differs from Sperry *et al.* [3] approach, which calculates the costs of its optimisation routine based on the *k_rc_* normalised in function of *k*_rcmax_ and the critical *k*_rc_ value (*k_rc,crit_*), where the cavitation costs (θ) are assumed to be maximum:

$\theta=\frac{k_{rc,max}-k_{rc}}{k_{rc,max} - k_{rc,crit}}$

In this study we assume *k_rc,crit_* = 0, similarly to Sperry *et al.* [3] which assumes *k_rc,crit_* is 0.005∙*k_rc,max_*. This assumption makes the cost function used in SOX (equation 8 in the main text), equivalent to *1-θ*. The main advantage of the SOX cost function is the possibility of carrying out the optimisation without the need for the *A* normalisation required in Sperry *et al.* [3], which eliminates the need of finding the instantaneous maximum *A* for each set of environmental conditions. Changes to the assumption that *k_rc,crit_* = 0 can also be easily introduced to SOX producing a more conservative stomatal behaviour (Fig. S2).

The differences between the structure and optimisation routine of our model and Sperry *et al.* [3] were made to simplify the model numerically, facilitating its integration in large-scale models. The model presented in this study is compatible with any photosynthetic model, does not make assumptions over canopy energy-balance and boundary layer calculations and, due to its simplicity, can be approximated analytically. The analytical approximation of our model will be described in future studies and will greatly facilitate the use of xylem hydraulics based stomatal optimisation schemes in large-scale modelling applications.

**Notes S4.** *Changes in soil hydraulic conductance*

Changes in soil hydraulic conductance can also be included in SOX by computing Ψ_r_ as a function of soil-to-root conductance expressed on a leaf area basis (*k_sr_*; mol m^-2^ leaf s^-1^):

$\Psi_{r}=\Psi_{s}-\frac{E}{k_{sr}}$

where Ψ_s_ is the Ψ of the bulk soil close to roots. Assuming *k_sr_* changes are dominated by soil hydraulic conductance changes we use Clapp & Hornberger [4] to model the relationship between *k_sr_* and Ψ_s_:

$k_{sr}=k_{sr,max}\left( \frac{\theta}{\theta_{s}} \right)^{2b+3}$

where *k_sr,_*_max_ is *k_sr_* at saturated soil, *b* is an empirically estimated parameter related with soil physical properties, θ is the soil volumetric water content (m^3^ m^-3^) and θ_max_ is the saturated soil θ, which can be related to Ψ_s_ as:

$\frac{\theta}{\theta_{s}}=\left( \frac{\Psi_{s}}{\Psi_{s,max}} \right)^{\frac{1}{-b}}$

where Ψ_s,max_ is the Ψ_s_ of the saturated soil. Most DGVMs already have soil hydrological schemes that compute Ψ_s_ and soil conductivity using Clapp & Hornberger or similar equations [5,6], and equation 10 would be compatible with any of these models. For calculating the Ψ_s_ over multiple soil layers, often simulated in DGVMs, the root fraction and soil moisture in each layer can be used to compute the weighted mean Ψ_s_ on the root zone, similarly to the approach currently employed in many DGVMs for computing the soil drought factor [5,6]

**Notes S5.** *Calculation of plant maximum hydraulic conductance*

The *k*_rc,max_ is calculated on a leaf area basis from maximum petiole level hydraulic conductivity, *K*_pet,max_ (mol m^-1^ s^-1^ MPa^-1^), the ratio between active xylem area and leaf area (i.e. Huber value, *h_v_;* m^2^ m^-2^), canopy height (*h*; m), and a unitless tapering factor (χ_tap_) to account for changes in conduit diameter within trees:

$k_{rc,max}=\frac{K_{pet,max}h_{v}}{h}\chi_{tap}$

The *K_pet,max_* is calculated from maximum branch xylem conductivity (*K_x,max_*; mol m^-1^ s^-1^ MPa^-1^) following Christoffersen et al [7]:

$K_{pet,max}=K_{x,max}\left( \frac{r_{int,pet}}{r_{int,ref}} \right)^{2}$

where *r_int,pet_* is the petiole conduit radius set to 10 µm and the *r_int,ref_* is the radius of conduits of the terminal branches set to 22 µm [8], to account for conduit tapering from branch to petiole. We define the hydraulic tapering factor (χ_tap_) as

$$\chi_{tap}=\frac{\chi_{tap:notap}(h)}{\chi_{tap:notap}(1)}$$

where χ_tap:notap_(*h*) and χ_tap:notap_(1) are factors that represents the ratio of the theoretical whole tree conductance with taper (*K_max,tap_*) to that without taper (*K_max,notap_*) at height *h* and 1 m, respectively. These factors are calculated following the Savage et al [9] model as described by Christoffersen *et al.* [8].

$$K_{max}=a\left( n_{ext}^{N/2} \right)^{b}$$

where *a* and *b* are constants set to 7.2 x10^-13^ and 1.32, to calculate *K_max,notap_* and 6.6 x10^-13^ and 1.85 for *K_max,tap_* [7]. The *n_ext_* represents the branching patterns in the Savage *et al.* [9] model and is set to 2. The *N* is the total number of branching levels, calculated as a function of *h*:

$$N=\frac{3ln\left[ 1-\frac{h}{L_{pet}}(1-n_{ext}^{1/3}) \right]}{ln(n_{ext})}-1$$

where *L_pet_* is petiole length set to 0.04 m [9].

**Notes S6.** *Description of the Unified Stomatal Optimisation model*

The Unified Stomatal Model (USO) from Medlyn et al [10] relates stomatal conductance to water (*g*_w,_ mol m^-2^ s^-1^) with *A* (µmol m^-2^ s^-1^) using two empirically-estimated parameters: the minimum *g*_w_, *g*_0_ (mol m^-2^ s^-1^), and *g*_1_ (kPa^-0.5^):

$g_{w}=g_{0}+1.6\left( 1+\frac{g_{1}}{\sqrt{D}} \right)\frac{A}{c_{a}}$

where *c*_a_ is in µmol mol^-1^ and *D* in kPa. The USO parameter *g_1_* can be related to the carbon cost of water as:

$g_{1}\propto\sqrt{\frac{\Gamma^{*}}{\lambda}}$

, where Γ^*^ is the CO_2_ compensation point in the absence of dark respiration (kPa). The USO parameters were computed from the modelled *A* and *g*_w_ by all the models evaluated against the Caxiuanã data in Figure 3.

**Supplementary Tables**

**Table S1.** Plant inputs used for the Caxiuanã National forest site

| **Symbol** | **Value** | **Units** | **Source** |
| --- | --- | --- | --- |
| ω | 0.15  2.9 x 10^-5^  43  13  0.08  190  1.2 x 10^-5^  30  -2.3 | - | [2] |
| *V*_cmax25_ |  | mol m^-2^ s^-1^ | [13] |
| *T*_upp_ |  | °C | [14] |
| *T*_low_ |  | °C | [7] |
| α |  | mol mol^-1^ | [7] |
| *K*_x,max_ |  | mol m^-1^ s^-1^ MPa^-1^ | Measured |
| *h*_v_ |  | m^2^ m^-2^ | Measured |
| *h* |  | m | [15] |
| *Ψ_50_* |  | MPa | Measured |
| *r* | 3 | m | [7] |

**Table S2.** Plant inputs used for the Manaus (MAN) and Tapajós (TAP) forest sites

| **Symbol** | **Value** | | **Units** | | **Source** |
| --- | --- | --- | --- | --- | --- |
|  | ***MAN*** | ***TAP*** |  |  | |
| *V*_cmax25_ | 4.3 x 10^-5^ | 2.9 x 10^-5^ | mol m^-2^ s^-1^ | [16,17] | |
| *h* | 35 | 35 | m | [18] | |
| *Ψ_50_* | -2.1 | -2.3 | MPa | [19,20] | |

*Note: The remaining plant inputs were assumed to be equal to the control plot in Caxiuanã site (Table S1).*

**Table S3.** Description of the simulations conducted at the three amazon sites

| **Simulation** | ***P_s_*** | ***T_a_* *&* *D*** |
| --- | --- | --- |
| Sim1 | Anomalies  Anomalies  No Anomalies  No Anomalies | Anomalies |
| Sim2 |  | No Anomalies |
| Sim3 |  | Anomalies |
| Sim4 |  | No Anomalies |

*Note: “Anomalies” refers to the default meteorological input from the 1900-2016 CRU-NCEP dataset. “No Anomalies” refers to the meteorological input without climatic anomalies, represented by an average year from the 1901-2016 CRU-NCEP dataset.*

**Table S4.** Parameters from the Unified Stomatal Optimisation model *(*USO) estimated using the SOX and *β*-models output from the simulations conducted at the control and throughfall exclusion (TFE) plots in the Caxiuanã National Forest (Fig. 3).

| **Model** | **Parameter** | **Control(±SE)** | **TFE(±SE)** |
| --- | --- | --- | --- |
| **SOX**  **(fixed *a*)** | *g_0_* | 0.09(±0.00) | 0.04(±0.00) |
|  | *g*_1_ | 5.74(±0.04) | 3.28(±0.01) |
| **SOX**  **(optimized *a*)** | *g_0_* | 0.05(±0.00) | 0.04(±0.00) |
|  | *g*_1_ | 4.57(±0.01) | 3.16(±0.01) |
| *β***_fun_** | *g_0_* | 0.00(±0.00) | 0.00(±0.00) |
|  | *g*_1_ | 3.80(±0.01) | 3.85(±0.00) |
| *β***_off_** | *g_0_* | 0.00(±0.00) | 0.00(±0.00) |
|  | *g*_1_ | 3.86(±0.01) | 3.86(±0.01) |

*Note:* *We use the SOX output with a fixed shape parameter (a=2.1) for equation 5 and with a shape parameter optimized for each plot (a=1.1 in control and a=2.54 in TFE).*

**Supplementary Figures**

**
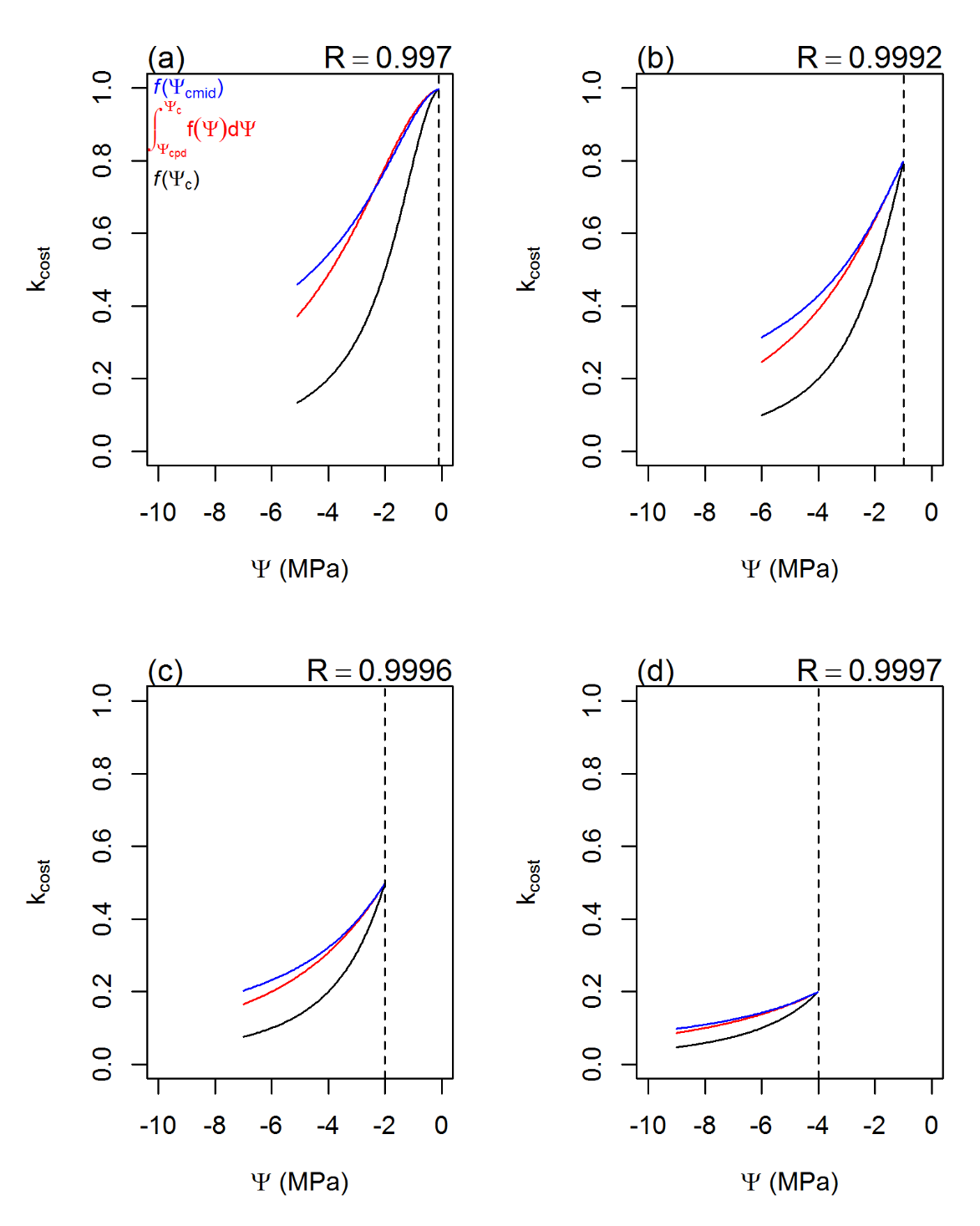
**

**Figure S1.** Comparison of equations 5 and 6 to compute the normalised root to canopy hydraulic conductance (*k_cost_*). Equation 5 was solved using either the canopy water potential (Ψ_c_, MPa) or the middle value of the root-canopy gradient (Ψ_c,mid_, see equation 7 in the main text). The dashed line is the pre-dawn Ψ_c_ (Ψ_c,pd_). Equation 6 was solved exactly assuming the shape parameter of the vulnerability curve (*a*) is 2 and the Ψ_50_ is -2 MPa. The correlation coefficient indicated on each panel refers to the agreement between the *k_cost_* calculated using *f*(Ψ_c,mid_) and equation 6.

**
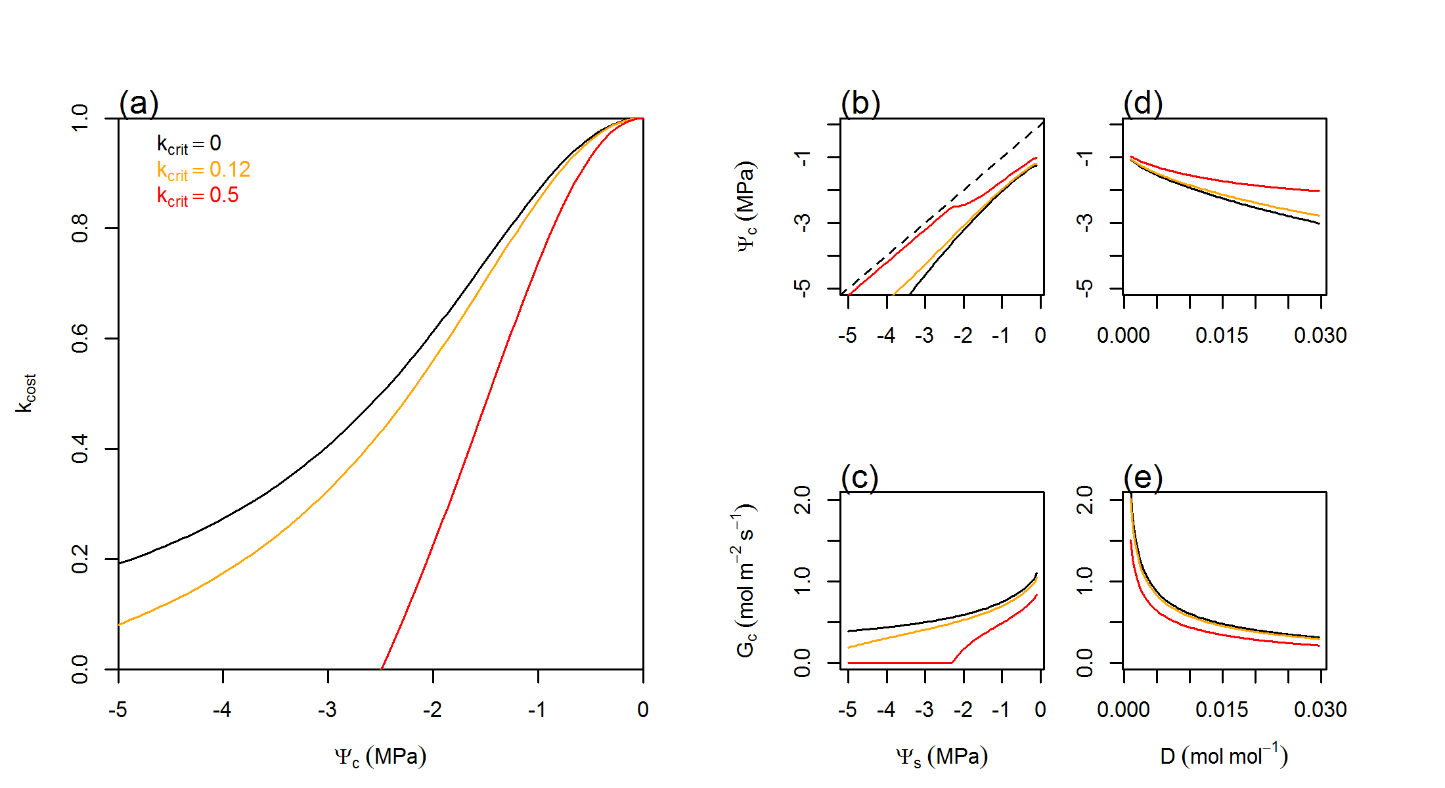
**

**Figure S2****.** Effect of different critical values of hydraulic conductance (*k_crit_*) for the computation of the normalised root to canopy hydraulic conductance (*k_cost_*). The default SOX formulation assumes *k_crit_* = 0 (black line), the yellow and red line indicate the model predictions assuming *k_crit_* = 0.12∙*k_rc,max_* or *k_crit_* = 0.5∙*k_rc,max_*. Plant and environmental inputs used in these simulations are given in Table 1.


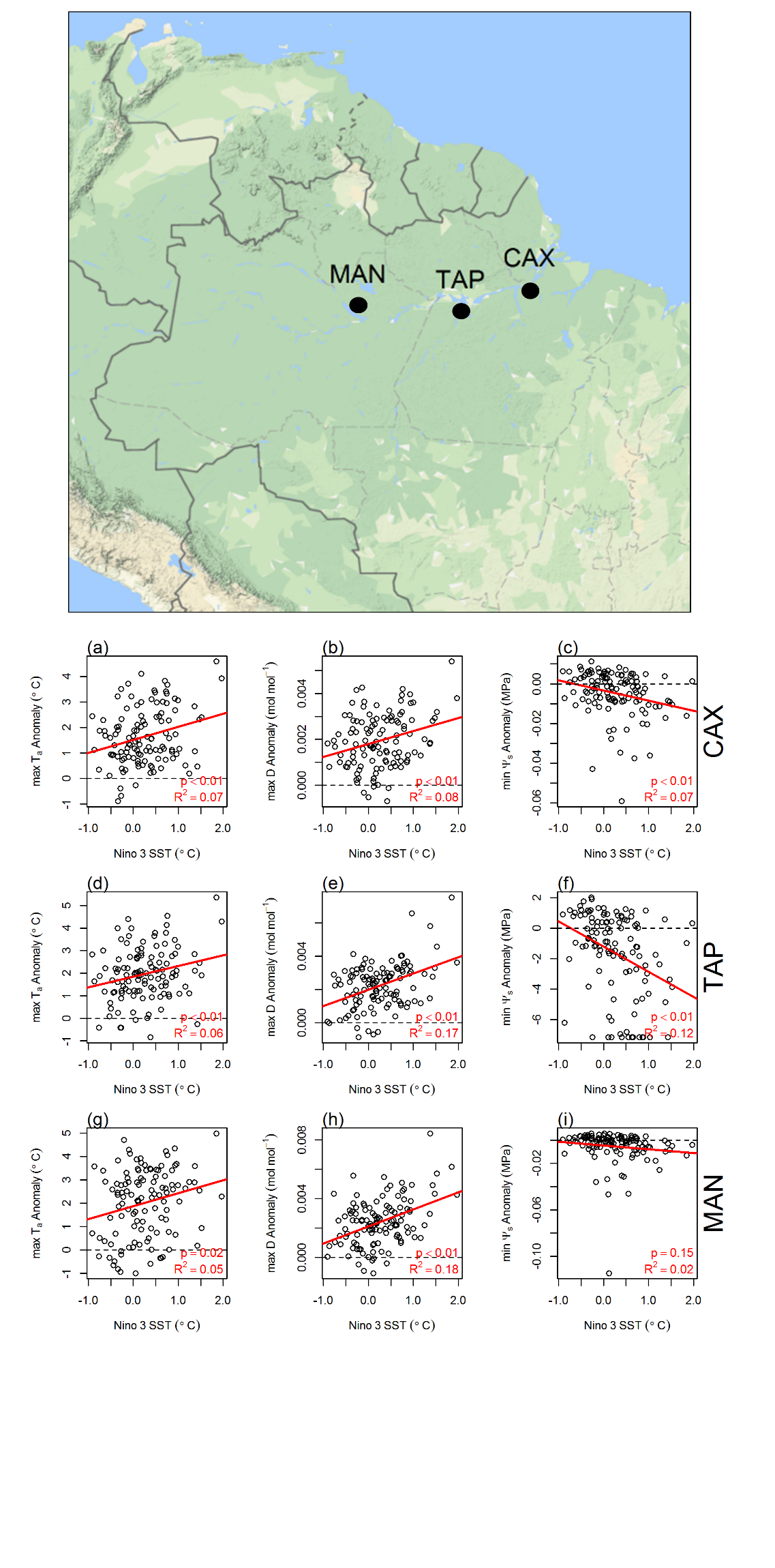
**Figure S3.** The three Amazon forest sites used in our study. The three sites are dominated by evergreen broadleaf *terra firme* tropical forest [21]. The Caxiuanã national forest site (CAX; 1°43’ S, 51°27’ W) has a mean annual precipitation of 2272 mm [15] land soils are predominantly clay-rich oxisols [21]. The Tapajós national forest site (TAP; 3°04’ S, 54°56’ W) has a mean annual precipitation of 2000 mm [22] and is located in a flat high plateau with a deep-water table and clay-rich oxisols interspersed with sandier patches [21]. The Manaus national forest site (MAN; 2°36’ S, 60°12’ W) has a mean annual precipitation of 2252 mm [23] and is located in a low plateau with clay-rich oxisols soil [21]. The panels below indicate the effect of sea surface temperature (SST) anomalies at the Niño 3 index location (5°N to 5°S and 150-90°W) on the maximum annual air temperature (T_a_), vapour pressure deficit (*D*) and minimum annual soil water potential (*P_s_*) at each site. The meteorological data is from the CRU-NCEP dataset, see text for details. Note the different y-axis scale on panels *c*, *f* and *i*.

**
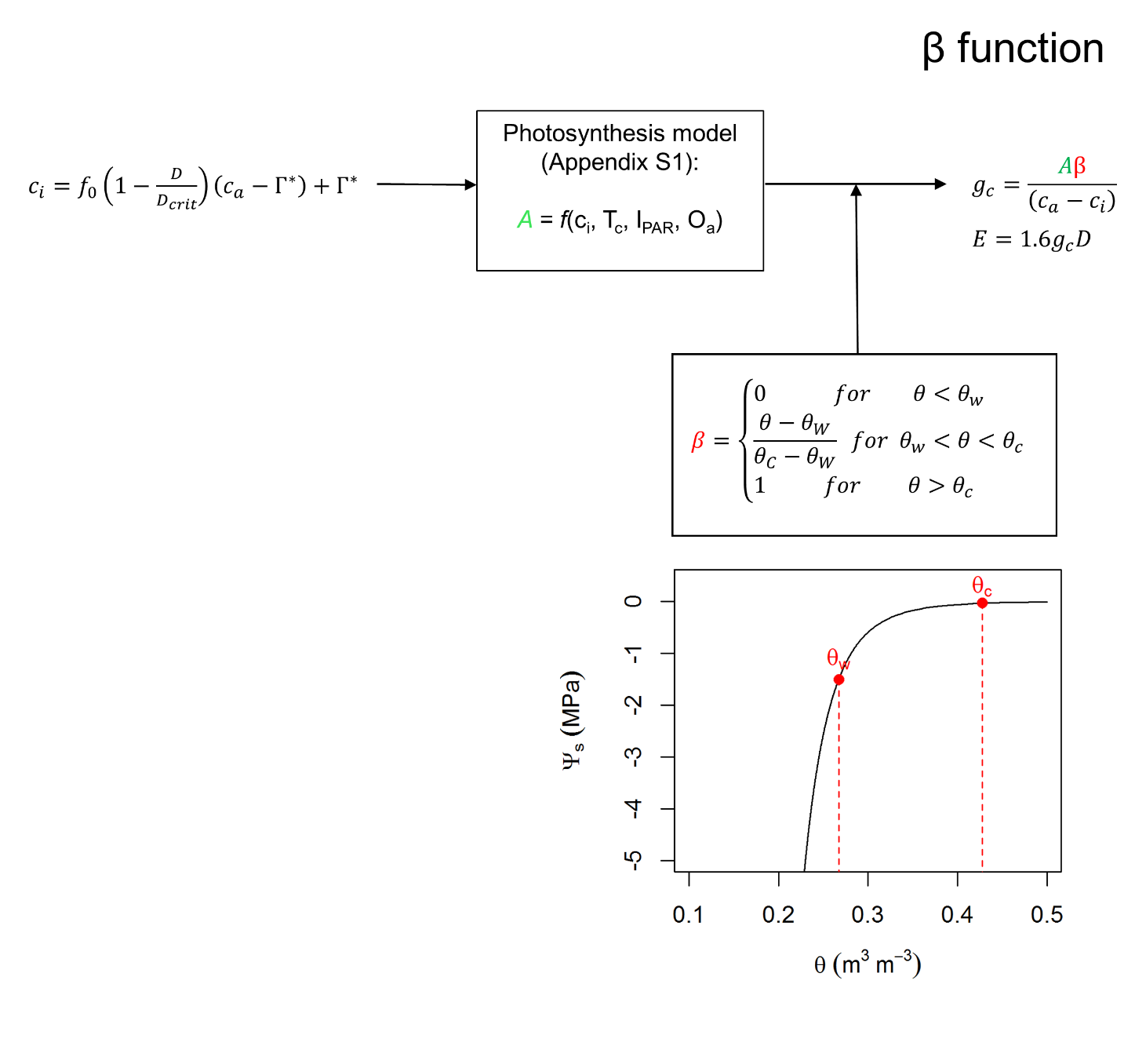
**

**Figure S4.** Description of the β-function model used in the study. This model uses the equation from Jacobs [24] to compute the leaf internal CO_2_ concentration (*c_i_*) used in the Collatz *et al.* [1] photosynthesis model described in Appendix S1. The carbon assimilation value (*A*) produced by the photosynthesis model is modified by the unitless drought factor β, which is computed as a function from soil moisture content (θ) and two parameters the critical (θ_c_) and wilting (θ_w_) points. The θ_c_ and θ_w_ represent the θ when *A* first falls below its unstressed value (i.e. the *A* produced by the Collatz model) and when *A* ceases completely, respectively. The θ_c_ corresponds to the θ when Ψ_s_ = -0.03 and θ_w_ to the θ when Ψ_s_ = -1.5 MPa [25]. The *β* parameter is multiplied by *A* to produce the soil moisture regulated *A*, which is then be used to calculate *g*_c_ and *E*. See Cox *et al.* [25] and the main text for details.

**
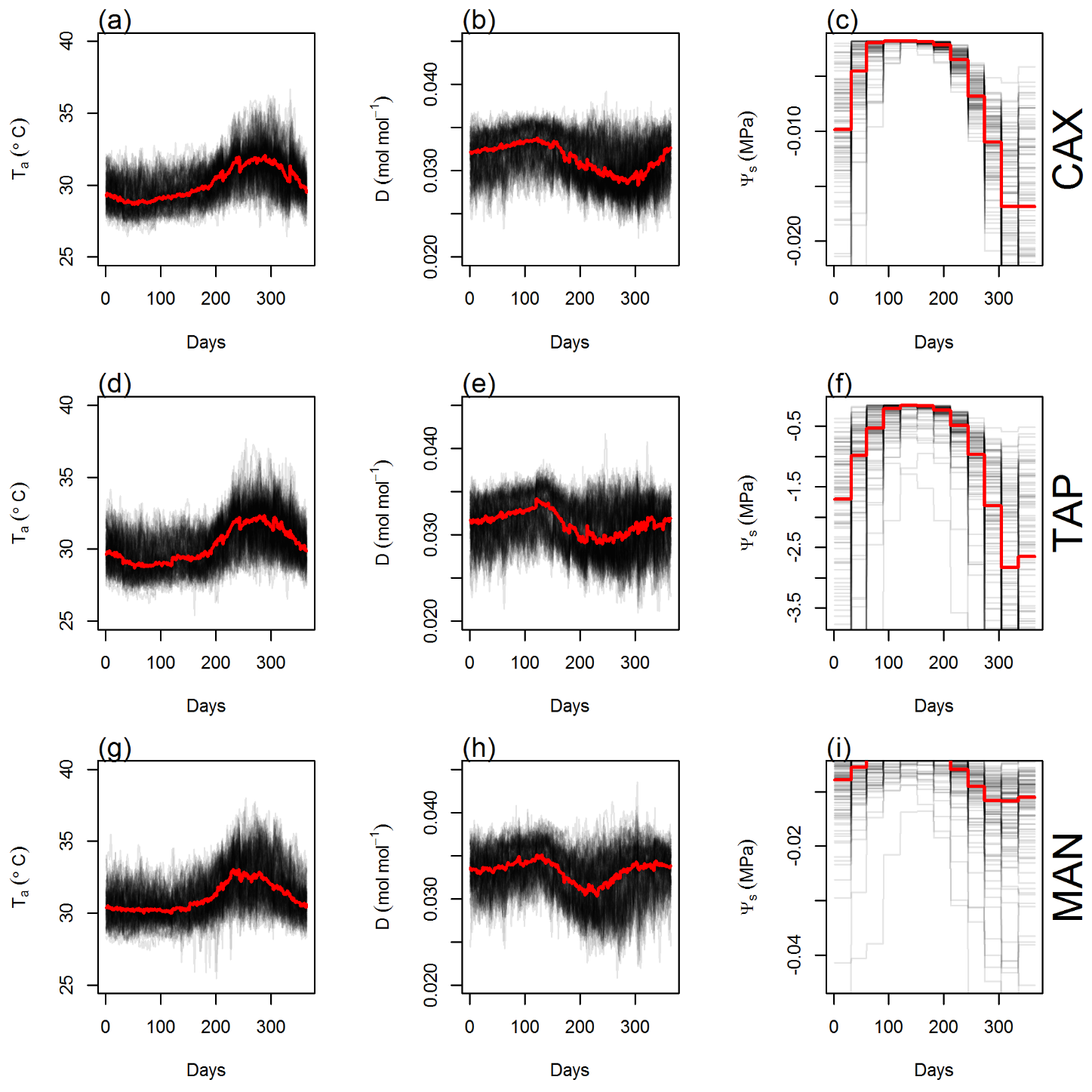
**

**Figure S5.** Environmental data used to drive the model for the long-term simulations for the three Amazonian sites used in this study, Caxiuanã (CAX), Tapajos (TAP) and Manaus (MAN). The lines represent the daily air temperature (*T_a_*), vapor pressure deficit (*D*) and soil water potential (Ψ*_s_*) at the midday from 1901 to 2016. The black lines are the unaltered meteorological data is derived from the 6-hourly CRU-NCEP dataset, while the Ψ*_s_* is the monthly soil moisture product from JULES simulations. The red lines correspond to the data without anomalies (i.e. an average year) used to drive Sim 2, Sim 3 and Sim 4.

**
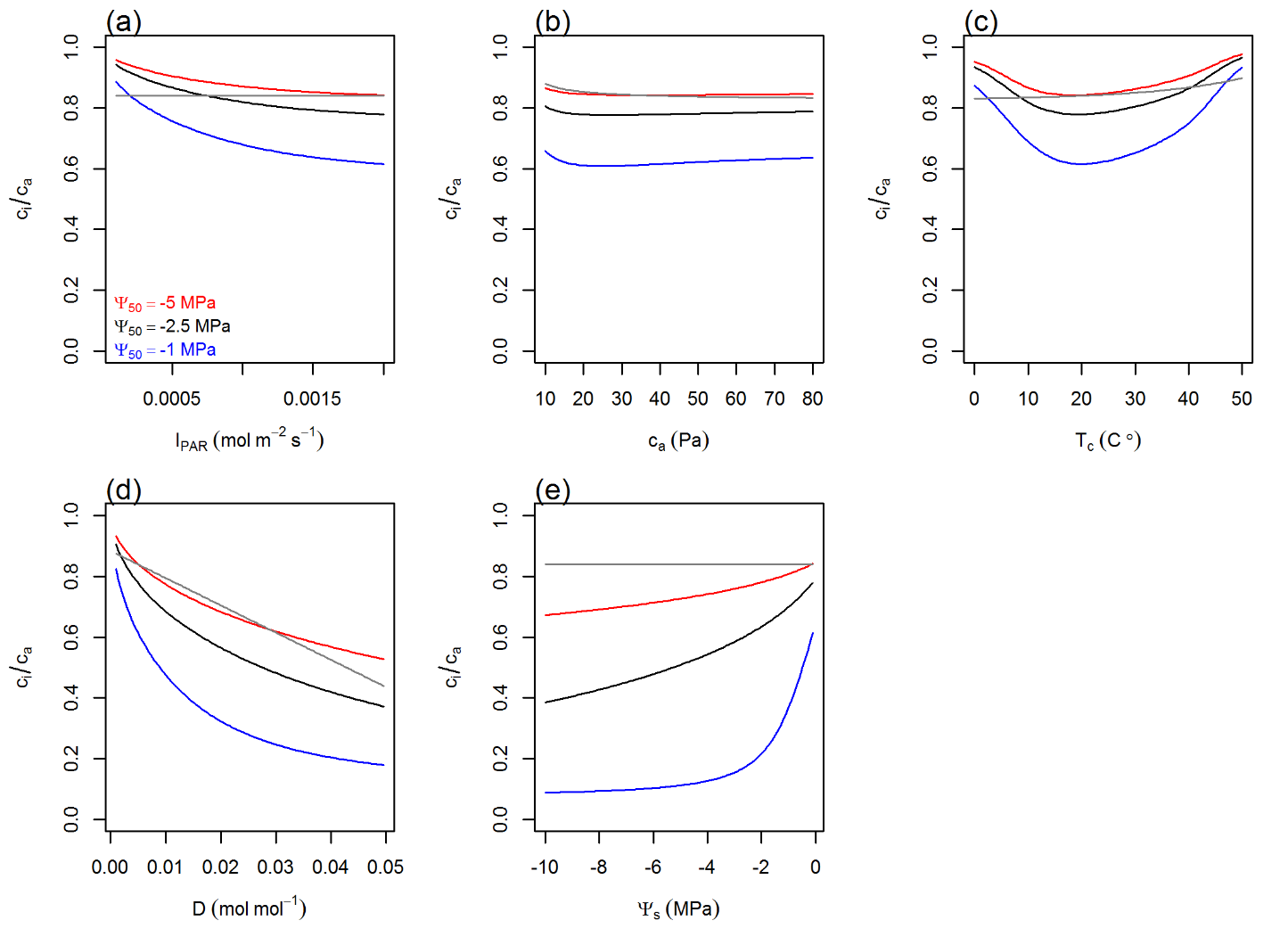
**

**Figure S6.** Theoretical relationships between intracellular to atmospheric CO_2_ concentration ratio (*c_i_/c_a_*) and shortwave irradiance (*I*_PAR_), atmospheric carbon partial pressure (*c*_a_), canopy temperature (*T_c_*), relative humidity (*D*), soil water potential (Ψ_s_), and vapour pressure deficit (*D*). All the other environmental and plant inputs were held constant at their default values (Table 1) except the parameters of the xylem vulnerability curve. The shape of the vulnerability curve (*a*) was calculated in function of the Ψ_50_, following Christoffersen *et al.* [7]. The grey line is the *c_i_* predicted by the Jacobs [24] equation used in the β model (Fig. S4).

**
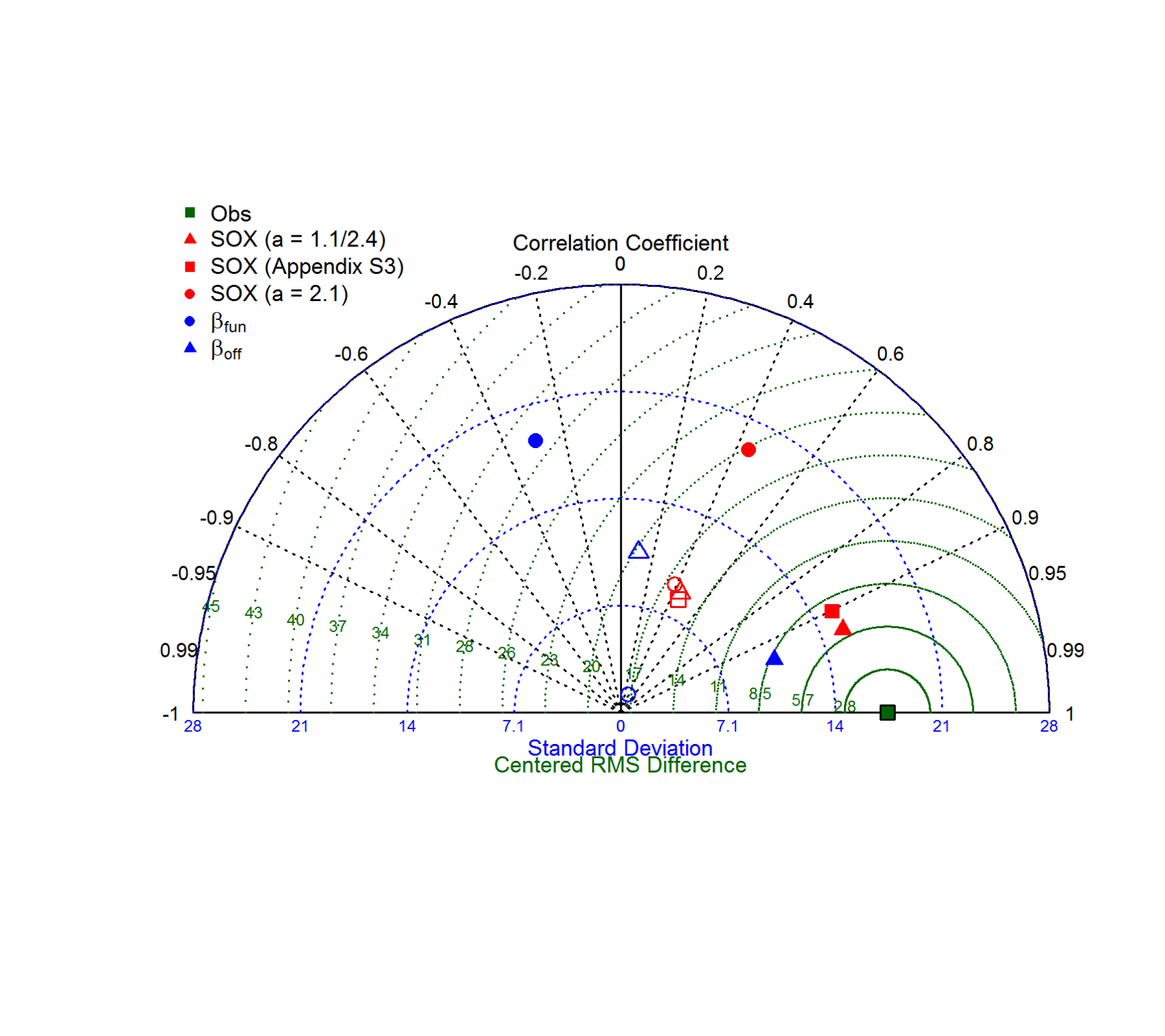
**

**Figure S7.** Taylor diagram using the monthly evaporation predictions for the Caxiuanã site. Points closer to the solid radial line have a similar standard deviation to the observations and the distance to the observations in the x-axis is proportional to the model centred root mean squared errors [26]. The filled symbols represent the control plot and the empty are the thoughfall exclusion treatment (TFE). The *β*_off_ is a model without an active representation of soil moisture stress (i.e. the model described in Fig. S4 with *β* set to 1).

**
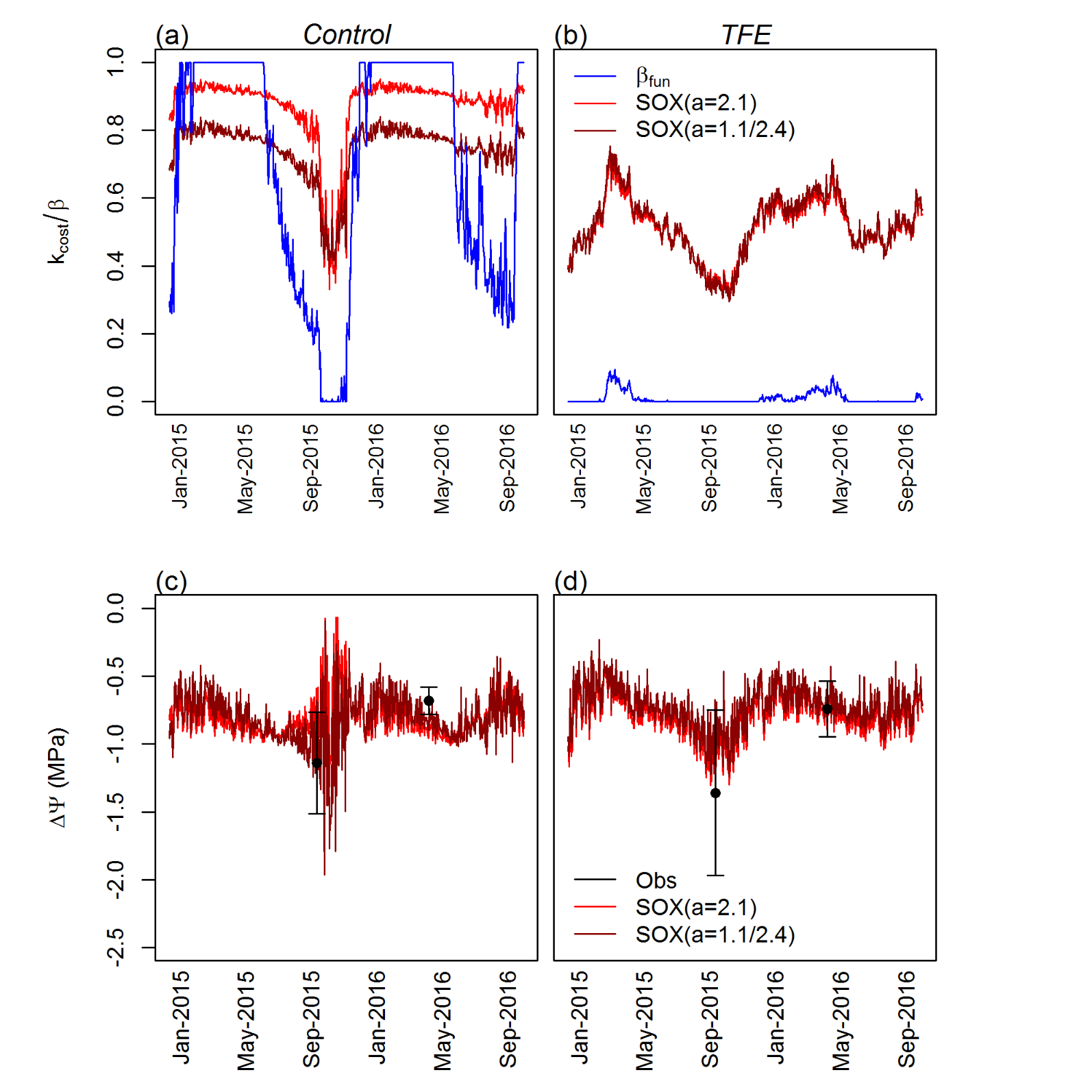
**

**Figure S8.** Minimum daily *β* values predicted by the β-function model and the normalised root to canopy hydraulic conductance (k_cost_) from SOX on the Caxiuanã forest simulation of the control (*a*) and throughfall exclusion (TFE, *b*) plots. The dark red line is the SOX with the shape of the vulnerability curve (*a*) optimised for each plot, whereas the light red line is the SOX with a single *a* for both plots computed from the Ψ_50_ following Christoffersen et al [7]. In the bottom panels we show the minimum daily canopy minus the predawn canopy water potential (ΔΨ*_c_*) simulated by SOX at the control (*c*) and TFE (*d*) plot. The black points are the mean water potential values measured at the site (midday Ψ_c_ – predawn Ψ_c_) and the bars are 2 x standard errors.

**
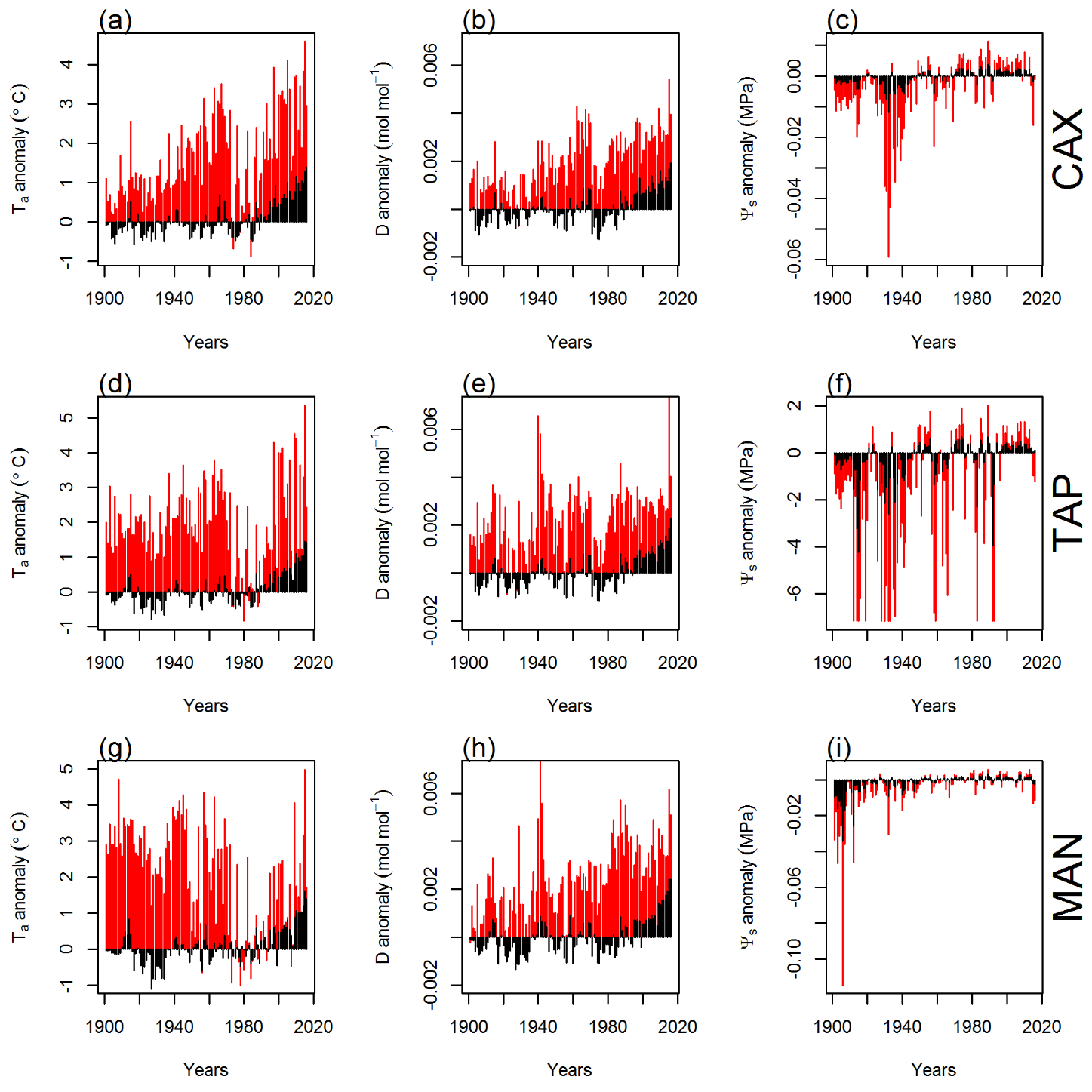
**

**Figure S9.** Anomalies in air temperature (T_a_) vapour pressure deficit (*D*) and soil water potential (Ψ_s_) at each study site, Caxiuanã (CAX), Tapajos (TAP) and Manaus (MAN). The black bars are the mean anomalies (i.e. difference between annual mean of each year and the mean of the entire dataset), the red bars are the maximum anomalies calculated as the difference between the T_a_ and D and minimum Ψ_s_ of each year and the maximum T_a_ and *D* and minimum Ψ_s_ of a year without climatic anomalies (i.e. the data used to drive Sim4 showed in Fig. S5).

**
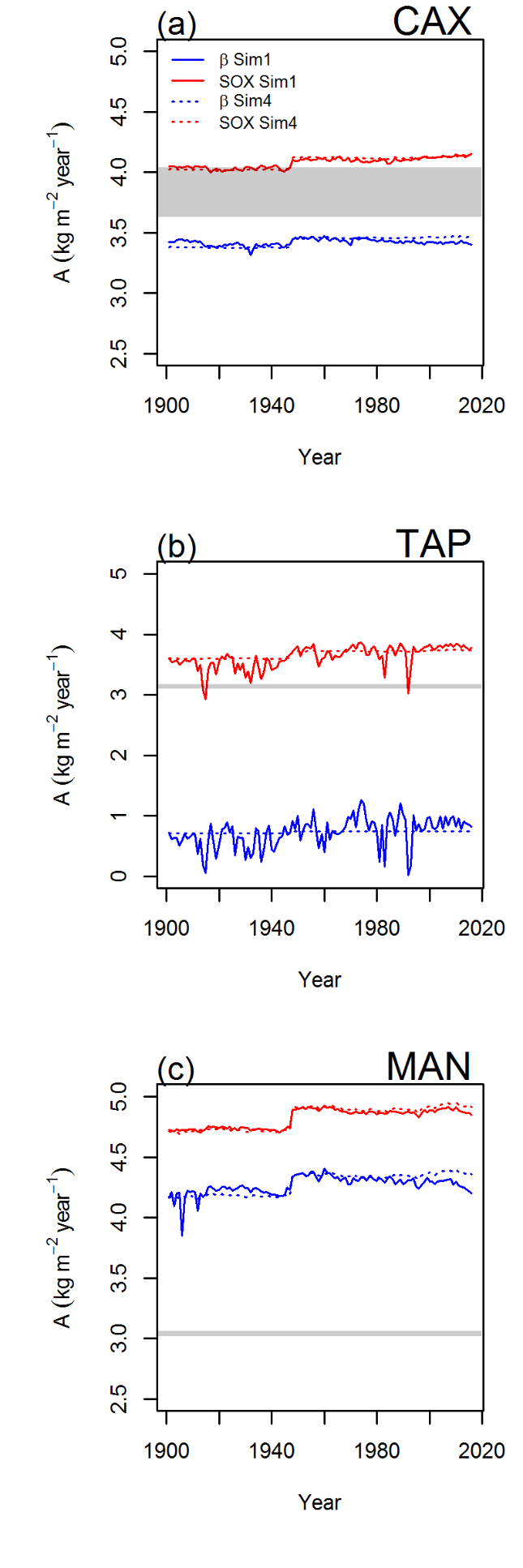
**

**Figure S10.** Annual canopy gross carbon assimilation predicted by SOX and by the β-function model (Fig. S4) using the default CRU-NCEP dataset (Sim1) and the driving data without climatic anomalies (Sim4) for the three sites used in this study, Caxiuanã (CAX), Tapajos (TAP) and Manaus (MAN). The grey bars are the interval of annual GPP values derived from eddy flux measurements taken from Malhi et al [21].

**References**

1. Collatz GJ, Ball JT, Grivet C, Berry JA. 1991 Physiological and environmental regulation of stomatal conductance, photosynthesis and transpiration: a model that includes a laminar boundary layer. *Agric. For. Meteorol.* **54**, 107–136. (doi:10.1016/0168-1923(91)90002-8)

2. Clark DB *et al.* 2011 The Joint UK Land Environment Simulator (JULES), Model description – Part 2: Carbon fluxes and vegetation. *Geosci. Model Dev. Discuss.* **4**, 641–688. (doi:10.5194/gmdd-4-641-2011)

3. Sperry JS, Venturas MD, Anderegg WRL, Mencuccini M, Mackay DS, Wang Y, Love DM. 2017 Predicting stomatal responses to the environment from the optimization of photosynthetic gain and hydraulic cost. *Plant. Cell Environ.* **40**, 816–830.

4. Clapp RB, Hornberger GM. 1978 Empirical equations for some soil hydraulic properties. *Water Resour. Res.* **14**, 601–604. (doi:10.1029/WR014i004p00601)

5. Best MJ *et al.* 2011 The Joint UK Land Environment Simulator (JULES), model description–Part 1: energy and water fluxes. *Geosci. Model Dev.* **4**, 677–699.

6. Oleson KW *et al.* 2004 Technical Description of the Community Land Model (CLM). *NCAR Tech. Note* (doi:10.5065/D6N877R0)

7. Christoffersen BO *et al.* 2016 Linking hydraulic traits to tropical forest function in a size-structured and trait-driven model (TFS v.1-Hydro). *Geosci. Model Dev.* **9**, 4227–4255. (doi:10.5194/gmd-9-4227-2016)

8. Friend AD. 1995 PGEN: an integrated model of leaf photosynthesis, transpiration, and conductance. *Ecol. Modell.* **77**, 233–255. (doi:10.1016/0304-3800(93)E0082-E)

9. Savage VM, Bentley LP, Enquist BJ, Sperry JS, Smith DD, Reich PB, Allmen EI Von. 2010 Hydraulic trade-offs and space filling enable better predictions of vascular structure and function in plants. (doi:10.1073/pnas.1012194108/-/DCSupplemental.www.pnas.org/cgi/doi/10.1073/pnas.1012194108)

10. Medlyn BE *et al.* 2011 Reconciling the optimal and empirical approaches to modelling stomatal conductance. *Glob. Chang. Biol.* **17**, 2134–2144. (doi:10.1111/j.1365-2486.2010.02375.x)

11. Zhou S, Medlyn B, Sabaté S, Sperlich D, Colin Prentice I. 2014 Short-term water stress impacts on stomatal, mesophyll and biochemical limitations to photosynthesis differ consistently among tree species from contrasting climates. *Tree Physiol.* **34**, 1035–1046. (doi:10.1093/treephys/tpu072)

12. Zhou S, Duursma RA, Medlyn BE, Kelly JWG, Prentice IC. 2013 How should we model plant responses to drought? An analysis of stomatal and non-stomatal responses to water stress. *Agric. For. Meteorol.* **182**–**183**, 204–214. (doi:10.1016/j.agrformet.2013.05.009)

13. Rowland L *et al.* 2015 After more than a decade of soil moisture deficit, tropical rainforest trees maintain photosynthetic capacity, despite increased leaf respiration. *Glob. Chang. Biol.* **21**, 4662–4672. (doi:10.1111/gcb.13035)

14. Harper AB *et al.* 2016 Improved representation of plant functional types and physiology in the Joint UK Land Environment Simulator (JULES v4.2) using plant trait information. *Geosci. Model Dev.* **9**, 2415–2440. (doi:10.5194/gmd-9-2415-2016)

15. Fisher RA, Williams M, da Costa AL, Malhi Y, da Costa RF, Almeida S, Meir P. 2007 The response of an Eastern Amazonian rain forest to drought stress: Results and modelling analyses from a throughfall exclusion experiment. *Glob. Chang. Biol.* **13**, 2361–2378. (doi:10.1111/j.1365-2486.2007.01417.x)

16. Carswell FE, Meir P, Wandelli E V., Bonates LCM, Kruijt B, Barbosa EM, Nobre AD, Grace J, Jarvis PG. 2000 Photosynthetic capacity in a central Amazonian rain forest. *Tree Physiol.* **20**, 179–186. (doi:10.1093/treephys/20.3.179)

17. Wu J. 2015 How do Amazonian Tropical Forest Systems Photosynthesize under Seasonal Climatic Variability: Insights from Tropical Phenology. The University of Arizona. .

18. De Gonçalves LGG *et al.* 2013 Overview of the large-scale biosphere-atmosphere experiment in amazonia data model intercomparison project (LBA-DMIP). *Agric. For. Meteorol.* **182**–**183**, 111–127. (doi:10.1016/j.agrformet.2013.04.030)

19. Brum M *et al.* 2018 Hydrological niche segregation defines forest structure and drought tolerance strategies in a seasonal Amazon forest. *J. Ecol.* (doi:10.1111/1365-2745.13022)

20. de V. Barros F. 2017 FUNCIONAMENTO HIDRÁULICO E VULNERABILIDADE À SECA DE DUAS FLORESTAS TROPICAIS. University of Campinas.

21. Malhi Y *et al.* 2009 Comprehensive assessment of carbon productivity, allocation and storage in three Amazonian forests. *Glob. Chang. Biol.* **15**, 1255–1274. (doi:10.1111/j.1365-2486.2008.01780.x)

22. Nepstad DC. 2002 The effects of partial throughfall exclusion on canopy processes, aboveground production, and biogeochemistry of an Amazon forest. *J. Geophys. Res.* **107**, 8085. (doi:10.1029/2001JD000360)

23. Araújo AC. 2002 Comparative measurements of carbon dioxide fluxes from two nearby towers in a central Amazonian rainforest: The Manaus LBA site. *J. Geophys. Res.* **107**, 8090. (doi:10.1029/2001JD000676)

24. Jacobs C. 1994 Direct impact of atmospheric CO2 enrichment on regional transpiration. Wageningen Agricultural University.

25. Cox PM, Huntingford C, Harding RJ. 1998 A canopy conductance and photosynthesis model for use in a GCM land surface scheme. *J. Hydrol.* **212**–**213**, 79–94. (doi:10.1016/S0022-1694(98)00203-0)

26. Taylor KE. 2001 Summarizing multiple aspects of model performance in a single diagram. *J. Geophys. Res. Atmos.* **106**, 7183–7192. (doi:10.1029/2000JD900719)
